# Effect of Si/Zr ratio on the catalytic behavior of Pt-Cu/Zr-SBA-15 in continuous methane dry reforming

**DOI:** 10.1039/d6ra02279k

**Published:** 2026-04-20

**Authors:** Mohammad Khalid, Norah A. M. Alsaif, S. Ganesan, T. Vinod Kumar, S. Padmanabhan, S. Mahalingam, Maha Awjan Alreshidi, P. Saravanan, Ahmed M. Fallatah, S. Jayakumar, Krishna Kumar Yadav, A. Subramani

**Affiliations:** a Department of Pharmaceutics, College of Pharmacy, King Khalid University Asir-Abha 61421 Saudi Arabia; b Department of Chemistry, Graphic Era (Deemed to be) University Dehradun-248002 India; c Physics Department, College of Science, Princess Nourah Bint Abdulrahman University Riyadh 11564 Saudi Arabia; d Department of Mechanical Engineering, Sathyabama Institute of Science and Technology Chennai India; e Department of Mechanical Engineering, Vels Institute of Science, Technology and Advanced Studies (VISTAS) Chennai India; f Department of Mechanical Engineering, Vel Tech Rangarajan Dr. Sagunthala R&D Institute of Science and Technology Chennai Tamil Nadu India; g Department of Mechanical Engineering, Sona College of Technology Salem India; h Department of Chemistry, College of Science, University of Ha'il Ha'il 81451 Saudi Arabia; i Department of Chemistry, St. Joseph's College of Engineering OMR Chennai 600119 India; j Department of Chemistry, College of Science, Taif University P.O. Box 11099 Taif 21944 Saudi Arabia; k Department of Chemistry, Jerusalem College of Engineering (Autonomous) Pallikaranai Chennai 600100 Tamil Nadu India; l Department of VLSI Microelectronics, Saveetha School of Engineering, Saveetha Institute of Medical and Technical Sciences (SIMATS), Saveetha University Chennai 602105 Tamil Nadu India; m Environmental and Atmospheric Sciences Research Group, Scientific Research Center, Al-Ayen University Nasiriyah Thi-Qar Iraq; n Department of Chemistry, Dwaraka Doss Goverdhan Doss Vaishnav College (Autonomous) (Affiliated to the University of Madras, Chennai) 833, Gokul Bagh, E.V.R. Periyar Road, Arumbakkam Chennai 600106 Tamil Nadu India subuchem71@gmail.com

## Abstract

Mesostructured SBA-15 supports with varying zirconium contents were synthesized through a single-step hydrothermal route by systematically adjusting the Si/Zr molar ratio. These Zr-incorporated mesoporous materials were subsequently employed as hosts for a bimetallic catalytic system, in which 5 wt% copper served as the primary active phase while platinum (0.5 wt%) acted as a promoter. The active metals were introduced using the incipient wetness impregnation technique, employing copper nitrate and chloroplatinic acid as precursors. The structural and surface characteristics of the synthesized supports and catalysts were systematically explored using powder XRD, BET analysis, NH_3_-TPD, TPR, HR-SEM, HR-TEM, and TGA. Catalytic activity was assessed in the dry reforming of methane under atmospheric pressure. Reactions were conducted with equimolar CH_4_ and CO_2_ feeds (1 : 1 ratio) operated under a GHSV of 36 000 mL g^−1^ h^−1^, between 400 and 800 °C. Structural analyses verified the efficient embedding of Zr atoms within the silica structure. Particularly, samples with Si to Zr ratios of 5 and 10 exhibited enhanced surface acidity while avoiding the crystallization of ZrO_2_ (anatase phase). Among the tested formulations, the Pt-Cu/Zr-SBA-15 catalyst possessing a Si to Zr ratio of 5 displayed the most promising performance. It achieved CH_4_ and CO_2_ conversion levels of 90% and 95%, respectively, with a favorable H_2_/CO ratio of 3.8 after six hours of continuous operation. The catalyst also demonstrated excellent resistance to sintering and minimized coke accumulation, highlighting its long-term stability. Post-reaction analyses confirmed negligible carbon deposition on the Zr-modified catalysts. The superior DRM performance was attributed to several synergistic factors: strong electronic and structural interactions between copper and platinum, fine dispersion of the active metal species, reinforced metal-support bonding, and an optimal balance between weak and strong surface acid sites was observed for the catalyst.

## Abstract 2

Pt–Cu/Zr–SBA-15 exhibits superior dry reforming performance, achieving high CH_4_/CO_2_ conversions (90–95%), enhanced H_2_/CO ratio (3.8), and excellent stability with minimal coke, due to strong metal–support interactions and optimal acidity balance.

## Graphical abstract

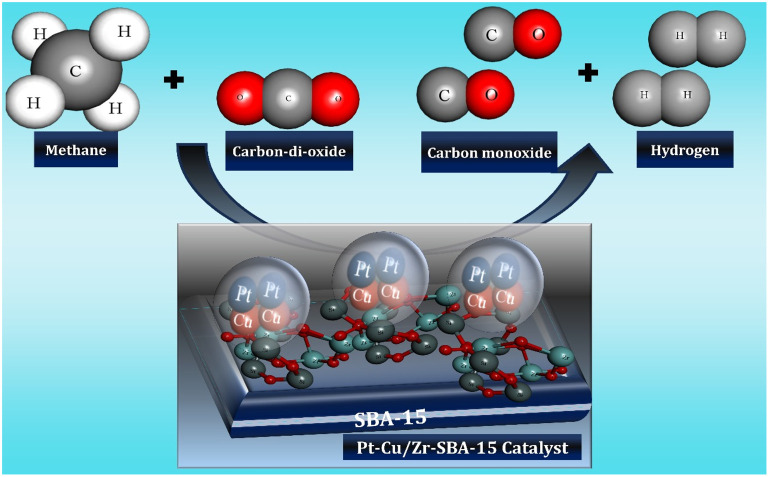

## 1. Introduction

Over the years, the depletion of fossil-based energy resources including coal, petroleum, and natural gas has become a growing concern. This decline is largely driven by the rapid expansion of the global population, which fuels increasing demands for transportation and industrial activities.^1^ In response to both the diminishing availability of these non-renewable resources and their negative environmental impacts, it is essential to explore cleaner and more sustainable energy alternatives.^2^ However, existing renewable energy sources may not be capable of fully meeting the rising global energy needs. As a result, researchers are actively exploring unconventional solutions to bridge this energy gap. One promising option is shale gas, an abundant source of natural gas, primarily methane found in underground rock formations across many regions of the world.^3–5^

When evaluating potential energy sources, it is crucial to assess their environmental consequences, particularly those linked to the use of non-renewable fuels.^6^ One of the most urgent environmental challenges facing the world today is the rapid progression of climate change and global warming, largely fueled by the emission of greenhouse gases. Among these, carbon dioxide and methane are major culprits, primarily originating from fossil fuel combustion and industrial processes.^7,8^ Methane, in particular, poses a significant threat due to its global warming potential estimated to be 28 to 36 times higher than that of carbon dioxide over a century making it a highly potent contributor to atmospheric warming.^9^ Additionally, methane is recognized as a precursor to ground-level ozone, another greenhouse gas with harmful environmental effects.^10^ Given these challenges, there is growing interest in transforming greenhouse gases into valuable chemical products as a sustainable and impactful mitigation strategy.^11^

Methane reforming is a highly regarded approach for transforming methane into valuable chemical intermediates, notably syngas (a mixture primarily of H_2_ and CO), which is crucial in numerous industrial processes.^12,13^ Synthesis gas (syngas) is a critical intermediate in the production of numerous industrial chemicals, including methanol, ethanol, dimethyl ether, hydrogen, and synthetic hydrocarbons through routes like Fischer–Tropsch synthesis.^14^ Various methods are employed for its generation, such as steam methane reforming (SMR), partial oxidation of methane, and dry reforming of methane (DRM).^15^ Among these, DRM has emerged as a promising approach owing to its economic feasibility and ability to utilize carbon dioxide and methane two major greenhouse gases as feedstocks, thus supporting both sustainability and efficiency goals.^16^ This method involves an endothermic reaction between methane and carbon dioxide to generate syngas.^17^ By fine-tuning reaction parameters and employing robust catalysts, hydrogen production can also be enhanced.^18^ However, one of the main difficulties in DRM lies in achieving long-term catalyst durability.^19^ Operating at high temperatures (typically between 700–900 °C), catalysts are prone to deactivation due to carbon deposition (coking) and thermal degradation, which negatively affect both conversion efficiency and product selectivity.^20^

To make dry reforming of methane (DRM) economically feasible, there is a strong focus on developing catalysts that do not rely on expensive noble metals.^21^ Transition metals like nickel (Ni),^22^ cobalt (Co),^23^ molybdenum (Mo),^24^ and copper (Cu)^25^ have emerged as promising candidates attributable to their low cost and reasonable catalytic efficiency.^26^ Although noble-metal-based catalysts, *e.g.*, rhodium (Rh), palladium (Pd), ruthenium (Ru), and platinum (Pt) are known for their superior catalytic efficiency, their high price limits large-scale applications.^27^ Among the transition metals, nickel stands out due to its considerable ability to convert methane and carbon dioxide, achieving results comparable to noble metal-based systems.^28^ However, nickel catalysts tend to suffer from thermal instability, primarily due to sintering and carbon deposition, which diminish their long-term effectiveness. To address these drawbacks, small amounts of noble metals like Pt, Rh, Pd, or Ru are often introduced as promoters.^29^ These additives help inhibit carbon accumulation and enhance the dispersion and stability of the active metal sites.^30,31^ Platinum, in particular, has proven highly effective when paired with nickel in bimetallic catalyst formulations. Their compatibility, rooted in similar atomic sizes and crystal structures as Group 10 elements, facilitates alloy formation, which contributes to improved catalytic properties.^32^ Studies have reported excellent DRM activity with Pt–Ni-based systems supported on various materials such as CeO_2_ (*e.g.*, Pt-Ni/CeO_2_,^33^ PteNi@CeO_2_ (ref. 34)) and hydrotalcite (*e.g.*, Pt-Ni/HT^35^).

Nickel is highly effective in activating methane to produce hydrogen but is prone to deactivation due to carbon (coke) build-up. Platinum, on the other hand, facilitates the dissociation of carbon dioxide and generates reactive oxygen species that help oxidize deposited carbon, thereby minimizing coke formation.^36^ When combined in a bimetallic Pt–Ni system, these metals work synergistically to lower the energy barriers for both methane activation and CO_2_ dissociation, enhancing overall catalyst performance and resistance to coking. The principal aim of the study is to create a cost-efficient catalyst by relying mainly on a non-noble metal. To enhance the durability and resistance to coke deposition, a small amount of platinum (approximately 0.5%) is incorporated as a promoter.^37^

Dry Reforming of Methane (DRM) is still far from large-scale commercialization, largely because conventional Ni-based catalysts struggle with thermal instability and rapid coke deposition. Numerous studies over time have aimed at overcoming this issue by combining nickel with other metals in an effort to improve its resistance to carbon build-up. While Pt–Ni supported catalysts have shown some promise, the problem of coke formation has not been fully resolved. Recognizing this limitation, recent research has shifted focus beyond just modifying the active metal itself. Increasing attention is now being given to tailoring the catalyst support, which serves a key role in improving coke resistance and overall catalyst durability.^38^

Catalytic activity is closely linked to surface area associated with the catalyst, which in turn is influenced by the effective dispersion of active metal species on the catalyst support. To improve both coke resistance and catalytic performance, various support materials have been explored. Supports such as SiO_2_, Al_2_O_3_, ZrO_2_, and a range of mixed-metal oxides have gained attention for their ability to promote better metal dispersion and reduce carbon deposition.^39,40^ These studies commonly highlight the impact of the interaction between metal and support in determining overall catalytic efficiency. Additionally, selecting the right support plays a critical role in optimizing syngas production, particularly in achieving a desirable H_2_/CO ratio.^41^

In recent developments, mesoporous silica frameworks, including MCM-41, SBA-15, TUD-1, and KIT-6 have emerged as effective catalyst supports due to their ability to enhance catalyst durability and tailor essential physicochemical features for reforming applications.^42^ These supports can be further engineered by *via* heteroatom incorporation, including Mg, Ce, Al, Ti, Fe, and Zr to modify characteristics such as surface acidity and porosity.^43^ Among them, SBA-15 stands out owing to its uniform pore structure and extensive surface area, and excellent thermal resilience, which stems from its thick pore walls and uniform channels. It also possesses low intrinsic acidity, making it suitable for various catalytic transformations. The Ni-loaded SBA-15 system has shown notable success in dry methane reforming, as highlighted by Ocsachoque *et al.*, who demonstrated that Ni/SBA-15 outperformed its Co-based counterpart in terms of catalyst performance and selectivity, suggesting its viability for industrial application.^44,45^ Further enhancement of SBA-15 catalysts can be achieved through isomorphic substitution, where silicon atoms in the framework are partially replaced by metal ions such as Zr^4+^ or Al^3+^.^46^ This substitution introduces new acid and redox sites Zr^4+^ incorporation, for instance, boosts Lewis acidity and can strengthen metal-support synergy, ultimately improving catalyst stability and overall reaction efficiency.^47^

In particular, the present work provides a systematic investigation of Pt–Cu bimetallic catalysts supported on Zr-modified SBA-15 with controlled Si/Zr ratios, aiming to understand how zirconium incorporation influences the physicochemical properties of the catalyst. By tuning the Si/Zr ratio, the study reveals how changes in surface acidity, metal dispersion, and metal-support interaction affect the catalytic behavior during DRM. This approach enables a clearer understanding of the structure-activity relationship between support modification and catalytic performance, particularly in terms of methane activation, CO_2_ utilization, and coke suppression. Such insights contribute to the rational design of mesoporous silica-based catalysts for efficient and stable DRM processes.

This study aims to explore the effect of incorporating zirconium into SBA-15 on the catalytic properties of Cu-containing catalysts promoted with platinum for the dry reforming of methane. The application of Zr-modified SBA-15 functioning as a catalyst support for Pt–Cu bimetallic catalysts in this reaction remains largely unexplored. This research also evaluates the catalyst's resistance to carbon deposition and its thermal stability, which are critical factors for practical application in greenhouse gas conversion. Although numerous studies have explored transition-metal catalysts for the dry reforming of methane, systematic investigations of bimetallic Pt–Cu catalysts supported on zirconium-modified SBA-15 with controlled Si/Zr ratios remain limited. The incorporation of Zr into mesoporous silica frameworks can significantly modify surface acidity, metal-support interactions, and structural stability, which are critical parameters influencing catalytic activity and coke resistance in DRM. In this context, the present study aims to elucidate the relationship between Si/Zr ratio, catalyst physicochemical properties, and catalytic performance in DRM. By tuning the Zr content within the SBA-15 framework, the study provides insight into how structural modification of the support affects metal dispersion, reducibility, acidity distribution, and catalytic behavior, thereby establishing meaningful structure-activity relationships for Pt-Cu/Zr-SBA-15 catalysts.

## 2. Catalytic activity

**Fig. 1:**
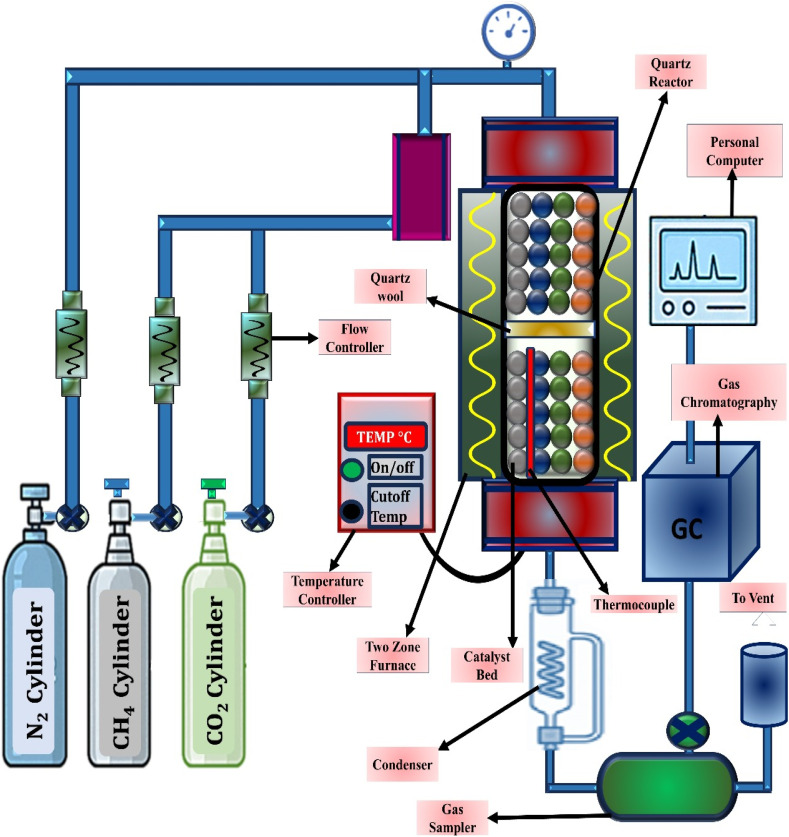
Schematic representation of the experimental setup for methane dry reforming.

The catalytic evaluation of the synthesized materials applied to the DRM was achieved in a stainless-steel continuous-flow fixed-bed reactor (High Tech Industries, Mumbai, India). Reactions were conducted under ambient pressure over a temperature window of 300–800 °C. In each run, approximately 200 mg of catalyst was packed into the reactor tube, confined by quartz wool plugs on both sides to secure the bed and maintain uniform flow distribution. A K-type thermocouple was inserted near the catalyst bed to accurately track the reaction temperature. Before exposure to the CH_4_/CO_2_ mixture, the catalysts underwent a pre-treatment step to ensure activation. The reduction was carried out in flowing hydrogen (20 mL min^−1^) at 700 °C for 2 h. Following reduction, the system was cooled to 300 °C, after which the reactant gases were introduced. The feed consisted of methane and carbon dioxide in a 1 : 1 molar ratio, with nitrogen serving as a diluent. The total inlet flow was controlled at 50 mL min^−1^, which corresponds to a GHSV of 1.0 mL g^−1^ h^−1^. Control experiments confirmed that non-catalytic components, such as quartz wool, did not contribute to methane or CO_2_ conversion under these conditions. The outlet gas stream was analyzed at regular intervals using a SHIMADZU GC-2014 analyzer configured with a thermal conductivity detector and a 2 m SHINCARBON ST packed column. Gas samples were injected *via* a six-port injection valve from VICI Valco fitted with a 1 mL capacity loop. A schematic representation of the experimental apparatus is depicted in Fig. 1.

The conversions of CH_4_ and CO_2_, the H_2_/CO product ratio, and the turnover frequency of methane (TOF(CH_4_)) were determined using the following calculations.1
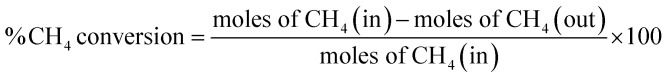
2
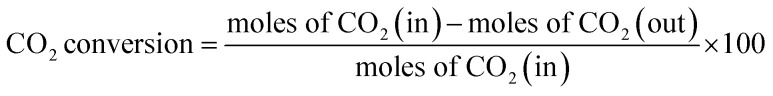
3
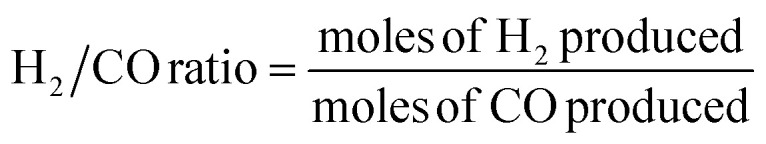
4
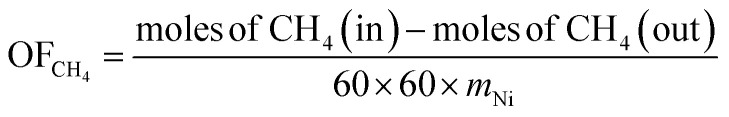


The molar quantities of methane and carbon dioxide introduced at the reactor inlet are denoted as CH_4_ in and CO_2_ in, respectively, while *n*CH_4_ out and *n*CO_2_ out represent their corresponding molar amounts at the reactor outlet. The term *m*_Cu_ refers to the number of moles of surface-exposed copper atoms.

### 2.1. Synthesis of SBA-15 and Zr/SBA-15

SBA-15 was fabricated with a mesoporous framework as a support material according to a hydrothermal route according to methods previously reported, employing TEOS as the silica precursor and Pluronic P123 as the structure-directing agent.^48^ Zirconium-modified SBA-15 samples with varying Si-to-Zr ratios were prepared through a one-pot direct hydrothermal approach, using zirconium oxychloride octahydrate as the zirconium precursor and TEOS as the silica precursor.^49^ For a representative synthesis, the P123 triblock copolymer (9.28 g) was introduced into 220 mL of double-distilled water, subsequently, 20 mL of 2.0 M HCl was introduced. This solution was agitated vigorously at 40 °C for 3 h to ensure complete dissolution. Subsequently, 22.78 g of TEOS together with the measured quantity of zirconium oxychloride octahydrate were introduced sequentially, maintaining Si to Zr ratios of 100, 50, 30, 20, and 10. The resulting mixture was stirred at 40 °C for 24 h before being subjected to a hydrothermal treatment (100 °C, 48 h) in a Teflon-coated autoclave. Filtration was used to separate the solid from the mixture, repeatedly rinsed with DI water, and being dried at 80 °C overnight in a hot-air oven. To eliminate the organic template, the dried sample underwent calcination in air at 550 °C for 6 h. The resulting mesoporous supports were designated as Zr-SBA-15(*x*), where *x* corresponds to the Si to Zr molar ratio (100, 50, 30, 20, or 10). The selected Si/Zr ratios of 5, 10, and 15 for catalytic evaluation were chosen to investigate a compositional gradient spanning framework Zr incorporation to potential extra-framework species formation, based on literature indicating structural transitions around these levels in mesoporous silicas. This range enables assessment of acidity enhancement and metal-support synergy impacts on DRM performance, with XRD confirming amorphous Zr integration up to Si/Zr = 5–15 (no anatase peaks), while narrower intervals (*e.g.*, 8–12) were deemed unnecessary given the gradual property evolution observed in our ICP-verified final ratios (5, 9, 14) and optimal activity at Si/Zr = 10.

### 2.2. Nickel and platinum metal loading on SBA-15 and Zr/SBA-15

**Scheme 1:**
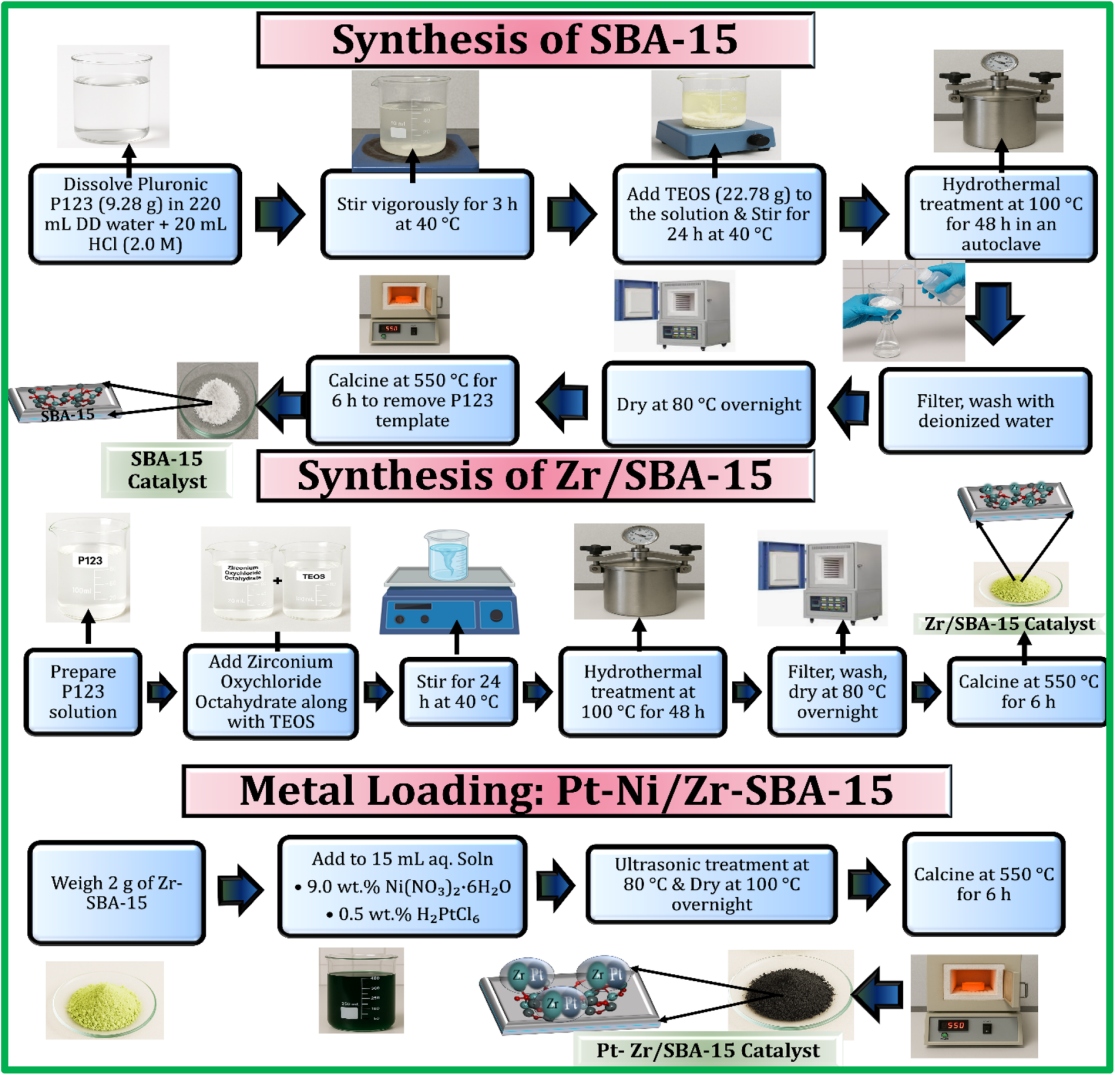
Shows the synthesis of Pt-Cu/Zr-SBA-15 catalyst.

Pt–Ni bimetallic catalysts deposited on Zr-modified SBA-15 were synthesized using the incipient wetness impregnation method (Scheme 1). In a typical preparation, 2 g of either SBA-15 or Zr-SBA-15 support was loaded with 15 mL of an aqueous precursor solution containing nickel nitrate hexahydrate and chloroplatinic acid, corresponding to metal loadings of 9.0 wt% Ni and 0.5 wt% Pt, respectively. The suspension was subjected to ultrasonic agitation at 80 °C until complete evaporation of the solvent was achieved. The solid product was dried at 100 °C for 12 h, then calcined at 550 °C for 4 h in air. For comparative purposes, an identical procedure was adopted for preparing Pt-Ni/SBA-15 without zirconium incorporation. The calcined catalysts were subsequently pelletized, followed by sieving to obtain 20–40 mesh particles, which were then employed in DRM catalytic activity tests.

### 2.3. Instrument used

X-ray diffraction (XRD) was performed on a Bruker D8 diffractometer with Cu Kα radiation (*λ* = 0.15418 nm) at 30 kV and 40 mA. Wide-angle patterns (2*θ* = 10–80°) were recorded with a 0.02° min^−1^ step size, and low-angle scans (0.6–5°) were used to examine mesoporosity. Textural properties were measured using a Quantachrome Autosorb (v3.0) after degassing samples at 300 °C for 3 h. N_2_ adsorption–desorption isotherms at −196 °C were analyzed by the BET method for surface area and BJH model for pore distribution. Temperature-programmed reduction (TPR) was carried out on ∼30 mg of calcined catalyst using 5% H_2_/He, with heating up to 900 °C at 10 °C min^−1^ after pre-treatment in He. Transmission electron microscopy (TEM) was performed on a JEOL JEM-2010 (120 kV) after dispersing powders in ethanol and depositing them on Cu grids. Thermogravimetric analysis (TGA) was conducted on ∼10 mg samples using a TA Q50 analyzer, heated from room temperature to 750 °C at 10 °C min^−1^ under air flow.

## 3. Results and discussion

### 3.1. XRD-analysis

**Fig. 2:**
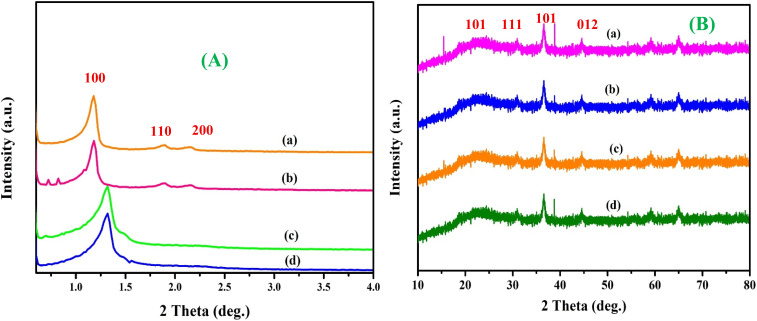
(A) Low-angle XRD patterns of (a) Pt-Cu/SBA-15, (b) Pt-Cu/Zr-SBA-15(5), (c) Pt-Cu/Zr-SBA-15(10), and (d) Pt-Cu/Zr-SBA-15(15); (B) high-angle XRD patterns of the corresponding catalysts.

Low-angle XRD measurements were carried out in the 2*θ* region of 0.5–5° to investigate the microstructural arrangement of SBA-15 and Zr-doped SBA-15 samples with varied ratio of Si and Zr. Fig. 2A depicting the diffraction pattern of the pristine SBA-15 material exhibited three distinct reflections at 2*θ* values of 1.11°, 1.65°, and 1.90°. The observed peaks can be indexed to the (100), (110), and (200) crystallographic planes, which are characteristic of the well-organized hexagonal mesostructure of SBA-15. These reflections observed are typical for a highly ordered hexagonal mesostructure with *P*6*mm* symmetry. Zr-modified SBA-15 materials exhibited comparable patterns, with diffraction peaks appearing at 0.98°, 1.56°, and 1.78°, indicating that the mesostructural order was retained following zirconium incorporation. A slight shift of peaks toward higher 2*θ* angles, especially evident in the Zr/SBA-15(5) sample (with peaks at 0.92°, 1.62°, and 1.70°), suggests a minor reduction in unit cell dimensions. This contraction is likely the result of Zr^4+^ ions being successfully integrated into the silica framework without causing significant structural disruption.^50–52^Fig. 2A. Low-angle XRD patterns of (a) Pt-Cu/SBA-15, (b) Pt-Cu/Zr-SBA-15(5), (c) Pt-Cu/Zr-SBA-15(10), and (d) Pt-Cu/Zr-SBA-15(15), showing characteristic reflections indexed to the (100), (110), and (200) planes of the hexagonal mesostructure (*P*6*mm* symmetry).

Fig. 2B. High-angle XRD patterns of the corresponding catalysts, where diffraction peaks at 2*θ* ≈ 37°, 43°, and 63° are indexed to the (101), (012), and (110) planes of CuO, while the peak at 2*θ* ≈ 39.8° corresponds to the (111) plane of PtO_2_. The presence of a peak near 2*θ* ≈ 25° is attributed to the (101) plane of anatase ZrO_2_ in samples with higher Zr content.

High-angle XRD analyses were performed to investigate the structural characteristics of the prepared catalysts, and the diffraction patterns are shown in Fig. 2B. For the Pt/Cu/Zr-SBA-15 catalyst with Si-to-Zr molar ratio of 100, no peak was observed around 2*θ* = 25°, indicating that bulk anatase ZrO_2_ did not form. In contrast, a noticeable diffraction peak near 2*θ* = 25° appeared in the samples with Si-to-Zr ratios of 20 and 10, confirming the presence of crystalline ZrO_2_ in the anatase phase. The absence of this peak at higher Si/Zr ratios suggests that zirconium was effectively incorporated into the silica framework, thereby preventing the formation of a separate ZrO_2_ phase. Furthermore, the presence of crystalline anatase became more pronounced with increasing Zr content. The observed peaks at diffraction angles 2*θ* of 37°, 43°, and 63° are attributed to the (101), (012), and (110) planes of CuO, indicating the presence of its cubic crystalline form. Well-defined CuO crystallites were particularly visible in samples with lower Zr content, while variations in Zr loading had little impact on the intensity or sharpness of these peaks. A peak at 2*θ* = 39.8°, attributed to the (111) plane of PtO_2_, was consistently observed in all Pt-Cu/Zr-SBA-15 catalysts.^53^

### 3.2. BET analysis

**Fig. 3:**
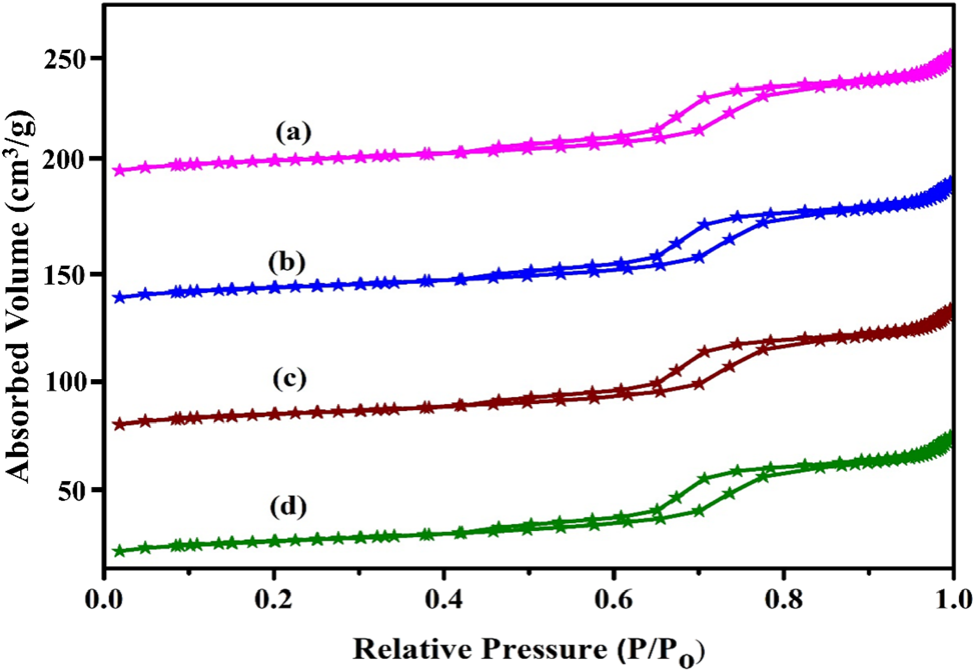
N_2_ adsorption–desorption isotherms of (a) Pt-Cu/SBA-15, (b) Pt-Cu/Zr-SBA-15(5), (c) Pt-Cu/Zr-SBA-15(10), and (d) Pt-Cu/Zr-SBA-15(15).

Fig. 3 illustrates the N_2_ adsorption–desorption measurements for both the pure SBA-15 as well as zirconium-modified samples of SBA-15. The unmodified SBA-15 exhibits a typical Type IV isotherm, featuring an H1-type hysteresis loop observed over the relative pressure interval 0.5–0.8, which is the indicative of well-ordered mesoporous materials with cylindrical pores undergoing capillary condensation. This confirms that the material possesses a highly ordered mesostructure. Interestingly, introducing zirconium into the SBA-15 framework did not change this fundamental feature. All Zr/SBA-15 samples, synthesized using varying Si to Zr ratios, displayed isotherm profiles almost identical to that of pristine SBA-15, indicating that the overall mesoporous architecture was preserved even after Zr incorporation. The textural data obtained from these isotherms are summarized in Table 1. These textural properties are expected to play an important role in determining the dispersion of the active metal species and consequently influence the catalytic performance of the Pt-Cu/Zr-SBA-15 catalysts in the DRM reaction. For the parent SBA-15, the specific surface area measured *via* BET was measured at 720 m^2^ g^−1^, exhibiting a pore volume of 0.79 cm^3^ g^−1^ and mean pore diameter of 4.0 nm. Upon zirconium incorporation, most samples exhibited an increase in surface area. This improvement can be linked to the expansion of the silica framework, as substituting smaller Si^4+^ ions with the larger Zr^4+^ ions causes slight distortion in the pore walls. Since the Zr–O bond (1.9 Å) is longer than the Si–O bond (1.58 Å), this substitution likely introduces microstrain and contributes to a subtle enlargement of the pore system. Among the modified variants, the Zr-SBA-15 sample with a Si to Zr molar ratio of 100 showed the most favorable textural characteristics highest surface area, porosity, and pore size suggesting that this composition allows for effective zirconium integration into the silica framework without substantial disruption of the mesostructure. However, further increases in Zr loading beyond this optimal ratio led to a progressive decline in BET surface area and total pore capacity. The metal composition and textural properties of the synthesized catalysts are summarized in Table 2. The Pt and Ni loadings determined by ICP-OES indicate that the actual metal content closely matches the nominal values, confirming efficient incorporation of the metals. The BET surface area and pore volume slightly decreased with increasing Zr content, suggesting partial pore blockage due to Zr incorporation. Additionally, the Cu particle sizes, calculated from XRD using the Scherrer equation, showed only minor growth after catalytic runs, indicating good structural stability (Table 2). This deterioration can be attributed to partial pore blockage caused by the formation of extra-framework zirconia species, particularly in the anatase phase, which hinder nitrogen diffusion and adsorption. Despite this, the hexagonal mesostructure remained largely intact even at higher Zr concentrations, highlighting the robustness of the SBA-15 framework against moderate metal incorporation.^54,55^

**Table 1:** Physicochemical properties and acidity of Pt-Cu/Zr-SBA-15 catalysts

Catalyst name	aSi/Zr molar ratio initial gel	aSi/Zr molar ratio final gel	b *S*_BET_ (m^2^ g^−1^)	c *V*_t_ (cm^3^)	d *D*_BJH_ (nm)	e *a*° (nm)	fWall thickness (nm)
Pt-Cu/SBA-15	—	—	754	0.712	5.23	11.2	6.2
Pt-Cu/Zr-SBA-15(5)	5	5	712	0.701	5.56	10.8	5.3
Pt-Cu/Zr-SBA-15(10)	10	9	689	0.689	5.56	10.2	5.2
Pt-Cu/Zr-SBA-15(15)	15	14	675	0.621	5.34	9.9	5.1

a The molar ratios of Si/Zr in the supports were determined using ICP-MS analysis.

b The specific surface area were determined using the BET method, with the BJH desorption technique applied for analysis.

c The specific pore volume distribution were determined using the BET method, with the BJH desorption technique applied for analysis.

d The specific pore size distribution were determined using the BET method, with the BJH desorption technique applied for analysis.

e The unit cell parameters and wall thickness were determined through low-angle X-ray diffraction (XRD) analysis and nitrogen sorption measurements.

f The unit cell parameters and wall thickness were derived from low-angle XRD patterns and N_2_ adsorption–desorption studies.

**Table 2:** Textural properties and metal dispersion of Pt-Cu/SBA-15 and Pt-Cu/Zr-SBA-15 catalysts

Catalyst name	aActual metal loading (wt%) Pt	aActual metal loading (wt%) Ni	b *S*_BET_ (m^2^ g^−1^)	c *V*_t_ (cm^3^)	c *D*_BJH_ (nm)	dCu particle size(nm)Fresh	dCu particle size(nm) spent
Pt-Cu/SBA-15	0.52	9.4	548	0.653	5.11	9.45	9.12
Pt-Cu/Zr-SBA-15(5)	0.48	9.37	521	0.612	5.02	9.37	9.27
Pt-Cu/Zr-SBA-15(10)	0.52	9.45	512	0.543	5.13	9.45	9.41
Pt-Cu/Zr-SBA-15(15)	0.49	9.23	501	0.512	5.06	9.23	9.20

a Elemental composition was analyzed *via* ICP-OES.

b The specific surface area was determined based on the BET theory.

c Calculated from the N_2_ desorption branch using the BJH method.

d Cu particle sizes for fresh and spent catalysts were calculated *via* XRD using the Scherrer formula.

### 3.3. NH_3_-TPD analysis of Pt-Cu/Zr-SBA-15 catalysts

**Fig. 4:**
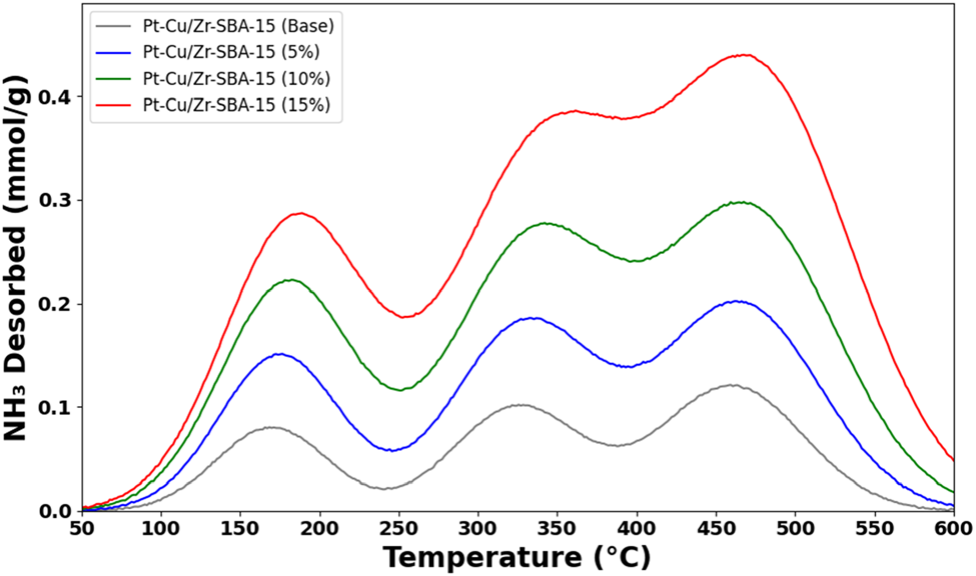
NH_3_-TPD profiles of (a) Pt-Cu/SBA-15, (b) Pt-Cu/Zr-SBA-15(5), (c) Pt-Cu/Zr-SBA-15(10), and (d) Pt-Cu/Zr-SBA-15(15).

Fig. 4 presents the NH_3_-TPD patterns of the Pt-Cu/Zr-SBA-15 catalysts, observed within the temperature window of 150–450 °C. The desorption patterns clearly highlight the distribution of acid sites with different strengths: ammonia release below 250 °C corresponds to weak acid sites, while desorption in the 250–450 °C region is associated with stronger acid sites. For the zirconium-free Pt-Cu/SBA-15 sample, the desorption signal was barely noticeable and restricted mainly to the weak acid region, indicating poor surface acidity. When zirconium was introduced into the SBA-15 framework, the overall acidity increased markedly, with the Pt-Cu/Zr-SBA-15(10) sample showing the highest value.^56^ However, further addition of zirconium reduced the acidity, most likely because of the formation of bulk crystalline anatase ZrO_2_, which contributes less effectively to surface acidity a trend also supported by the data in Table 3. Across all samples, weak acid sites dominated, probably arising from surface silanol (Si–OH) groups. Notably, the Pt-Cu/Zr-SBA-15(5) catalyst displayed a higher share of strong acid sites compared to the others. This can be attributed to the presence of well-dispersed Lewis acidic ZrO_2_ species anchored on the mesoporous surface. Considering the measured acidity, the catalysts follow the sequence: Pt-Cu/Zr-SBA-15(5) > Pt-Cu/Zr-SBA-15(10) > Pt-Cu/Zr-SBA-15(15) > Pt-Cu/SBA-15.

**Table 3:** Acid site distribution, hydrogen consumption, and carbon deposition of the catalysts evaluated using Pt-Cu/SBA-15, Pt-Cu/Zr-SBA-15(5), Pt-Cu/Zr-SBA-15(10), and Pt-Cu/Zr-SBA-15(15) catalyst^a^

Catalyst name	bReduction temperature (°C)	cVolume of H_2_ consumption (mmol g^−1^)	bCu loading (%)	dAcidity (mmol NH_3_ g cat^−1^) WA	dAcidity (mmol NH_3_ g cat^−1^) SA	dTotal acidity (mmol NH_3_ g cat^−1^)	eCarbon formation (%)
Pt-Cu/SBA-15	526, 600 700	368	9.45	80	67	157	65
Pt-Cu/Zr-SBA-15(5)	596	412	9.35	94	126	220	43
Pt-Cu/Zr-SBA-15(10)	547	454	9.23	125	135	260	22
Pt-Cu/Zr-SBA-15(15)	597	432	9.12	105	140	245	16

a SA = strong acid sites; WA = weak acid sites.

b Cu dispersion was calculated assuming a Had_{ad}ad/Cusur_{sur}sur ratio of 1.

c Hydrogen temperature-programmed reduction (H_2_-TPR) technique.

d Ammonia temperature-programmed desorption (NH_3_-TPD) technique.

e Carbon accumulation was measured *via* TGA.

Overall, these results emphasize that zirconium incorporation serves a fundamental role in modulating both the strength and distribution of acid sites in SBA-15-based catalysts.^57^ Such variations in surface acidity can significantly influence the adsorption and activation of reactant molecules, particularly CO_2_, thereby affecting the catalytic activity and stability of the catalysts during the DRM reaction. While NH_3_-TPD quantifies total acidity and site strength distribution, it does not differentiate Lewis from Brønsted sites. The observed strong acid sites (desorption 250–450 °C, peaking at 126–135 µmol g^−1^ for Si/Zr = 5–10) are attributed to framework Zr^4+^–O–Si Lewis acid centers, as extra-framework ZrO_2_ anatase (absent per XRD up to Si/Zr = 15) typically shows weaker acidity. This assignment aligns with enhanced CO_2_ activation in DRM (H_2_/CO = 3.8, minimal coking) and literature confirming Lewis acidity dominance in Zr-mesoporous silicas, where Py-IR routinely shows characteristic 1450–1600 cm^−1^ bands for coordinated pyridine on Zr^4+^ sites.

### 3.4. High-resolution SEM (HR-SEM) analysis

Fig. S1. presents the HR-SEM images of Pt-Cu/SBA-15, Pt-Cu/Zr-SBA-15(5%), Pt-Cu/Zr-SBA-15(10%), and Pt-Cu/Zr-SBA-15(15%) catalysts, illustrating the surface morphology and structural changes of the materials upon zirconium incorporation. Fig. S1a. corresponding to Pt-Cu/SBA-15 exhibits a well-defined, uniform, and porous structure, characteristic of the ordered SBA-15 support. The metal nanoparticles (Pt and Cu) appear relatively well dispersed over the surface, with minor agglomeration, indicating good initial dispersion. Upon introduction of zirconium (Zr), distinct morphological changes are observed. In Fig. S1b. Pt-Cu/Zr-SBA-15(5%), the surface becomes noticeably rougher, with a modest increase in particle clustering compared to the non-modified sample. This suggests initial structural rearrangement and interaction between Zr species and the metal-support system. As the Zr content increases to 10% in Fig. S1c. Pt-Cu/Zr-SBA-15(10%), particle agglomeration becomes more pronounced, and surface roughness intensifies. This reflects enhanced metal-support interaction, likely due to Zr acting as a structural stabilizer, promoting stronger anchoring of metal nanoparticles and potentially contributing to improved thermal stability. In Fig. S1d. Pt-Cu/Zr-SBA-15(15%), the surface morphology becomes highly heterogeneous, with large agglomerated structures and irregular features. This suggests significant structural modification, possibly due to the formation of zirconia-rich domains and metal cluster growth at higher Zr loadings. Overall, the HR-SEM results demonstrate that increasing the Zr content systematically alters the surface morphology from uniform and porous to rough and aggregated. These changes indicate improved metal-support interactions and may contribute to enhanced catalytic performance, thermal stability, and resistance to metal sintering under reaction conditions.

### 3.5. TEM analysis

**Fig. 5:**
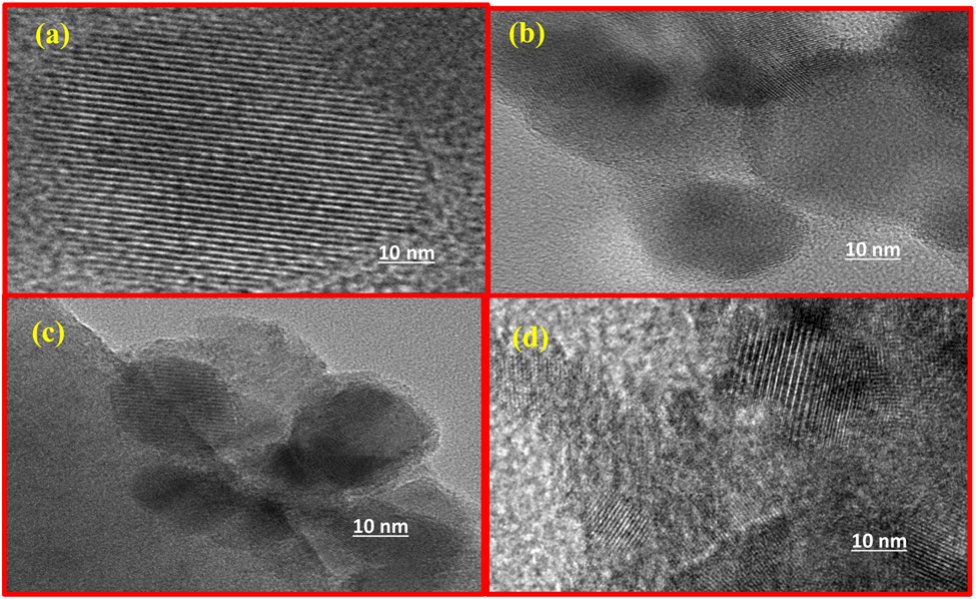
TEM images of (a) SBA-15, (b) Zr-SBA-15, (c) Pt-Cu/Zr-SBA-15(10), and (d) Pt-Cu/Zr-SBA-15(15).

Fig. 5 illustrates TEM micrographs of Pt-Cu/Zr-SBA-15 (10) catalysts captured at multiple magnifications. The pristine SBA-15 support exhibited a highly ordered mesoporous framework showing a distinctive hexagonal (honeycomb-arrangement) arrangement, as depicted in Fig. 5a. Upon incorporation of zirconium and subsequent deposition of copper and platinum, the mesostructure remained largely unaffected, retaining its periodic pore ordering without any discernible signs of particle agglomeration, as evidenced in Fig. 5b–d. TEM examination indicated that the Cu/Zr-SBA-15(10) catalysts possessed crystallite sizes predominantly within the 15–20 nm range. To obtain a more quantitative understanding of the metal dispersion, the particle sizes of Cu-based nanoparticles were estimated from several TEM images. The analysis indicated that the metal particles were predominantly distributed within the range of approximately 15–20 nm, with only a few larger agglomerates observed. The relatively narrow particle size distribution suggests effective dispersion of the active metal species on the mesoporous support, which can be attributed to the strong metal-support interaction promoted by zirconium incorporation into the SBA-15 framework. Such improved dispersion of active sites is expected to facilitate methane activation and contribute to the enhanced catalytic performance observed in the DRM reaction. However, occasional larger CuO domains were also observed. The introduction of a minor amount of platinum significantly improved the dispersion of copper oxide, likely due to enhanced metal-support interactions facilitated by the bimetallic interface. Furthermore, the presence of zirconium appeared to play a crucial role in mitigating particle sintering and promoting a more uniform arrangement of metal particles throughout the mesoporous architecture. The preservation of the ordered pore structure in the Pt-Cu/Zr-SBA-15 catalysts was consistently observed across all samples and was further validated through complementary low-angle XRD patterns and BET surface area measurements.^58–60^

### 3.6. H_2_-TPR analysis

**Fig. 6:**
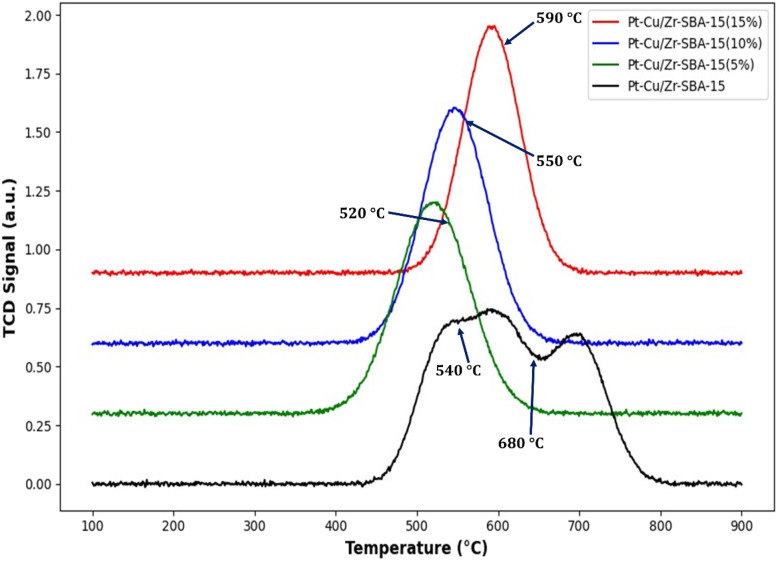
H_2_-TPR profiles of (a) Pt-Cu/SBA-15, (b) Pt-Cu/Zr-SBA-15(5), (c) Pt-Cu/Zr-SBA-15(10), and (d) Pt-Cu/Zr-SBA-15(15).

H_2_-TPR was employed to examine the reduction properties of Pt-Cu/SBA-15 catalysts with different Si/Zr ratios. The corresponding profiles are presented in Fig. 6, and the hydrogen consumption values are listed in Table 3. Measurements were carried out between 450 and 800 °C under hydrogen flow. For the Pt-Cu/SBA-15 catalyst, three distinct reduction peaks were observed at around 510, 600, and 700 °C. These reduction features can be attributed to the sequential reduction of different metal oxide species present on the catalyst surface. The peak appearing at lower temperature is associated with the reduction of PtO_*x*_ species to metallic Pt, whereas the peaks at higher temperatures correspond primarily to the stepwise reduction of CuO to Cu^0^. As the reduction temperature increases, changes in the oxidation state of copper species may occur, and the corresponding reduction signals can partially overlap with those of PtO_*x*_ species. Such overlapping reduction behavior is typical in bimetallic systems and indicates strong interaction between Pt, Cu, and the support. In addition, the incorporation of zirconium into the SBA-15 framework modifies the electronic environment of the active metals and enhances metal-support interactions, which can shift the reduction temperatures and broaden the TPR peaks. The broad reduction profile observed for the Zr-modified catalysts therefore suggests improved dispersion of the metal species and stronger anchoring of Pt and Cu particles on the support surface. The first peak was attributed to the reduction of surface PtO_2_/PtO species, while the second peak was assigned to the reduction of CuO to metallic Cu. The incorporation of zirconium altered the reduction behavior of the catalysts, leading to the appearance of a single reduction peak. This effect can be ascribed to improved dispersion of Pt and Zr over the SBA-15 surface through isomorphic substitution, which caused the reduction signals of Pt and Cu to overlap. For the bimetallic catalysts, the highest hydrogen uptake occurred between 400 and 700 °C. In the case of Zr-containing systems, a single broad peak was observed, attributed to the strong interaction of CuO and PtO with the Zr-SBA-15 support, which shifted the reduction temperature range to 450–800 °C for Si/Zr ratios between 5 and 15. An increase in Zr loading resulted in a larger peak area, reflecting higher hydrogen consumption. The merging of reduction peaks in Zr-containing catalysts indicates stronger metal-support interactions and explains the shift of reduction profiles toward lower temperatures. No separate Zr-related peaks were identified in the H_2_-TPR spectra of Cu-Pt/Zr-SBA-15 catalysts. The data confirm that the reducibility of the active metal species was enhanced in the presence of Zr-SBA-15 supports. Improved reducibility and stronger metal-support interaction can facilitate the formation of active metallic sites under reaction conditions, which is beneficial for methane activation and syngas production during DRM. Hydrogen consumption values (Table 3) further support this observation: catalysts supported on pure SBA-15 required only small amounts of hydrogen, whereas Zr-incorporated SBA-15 (Si/Zr = 10) exhibited the highest uptake. This increased hydrogen demand demonstrates fine dispersion of active metals and strong metal-support interactions in the Zr-modified catalysts. The combined results obtained from BET, NH_3_-TPD, H_2_-TPR, and TEM analyses provide valuable insight into the structure–activity relationships of the Pt-Cu/Zr-SBA-15 catalysts. The incorporation of zirconium into the SBA-15 framework modifies the surface acidity and strengthens the interaction between the active metal species and the support. Catalysts with moderate Zr incorporation exhibit improved dispersion of Cu and Pt species and enhanced reducibility, which facilitates the activation of methane and carbon dioxide during DRM. Furthermore, the increased Lewis acidity associated with Zr sites promotes CO_2_ adsorption and activation, contributing to higher catalytic activity and reduced carbon deposition. These results indicate that the catalytic performance is strongly dependent on the balance between metal dispersion, acidity distribution, and metal-support interaction, which is optimized at intermediate Si/Zr ratios.

### 3.7. TGA-analysis

**Fig. 7:**
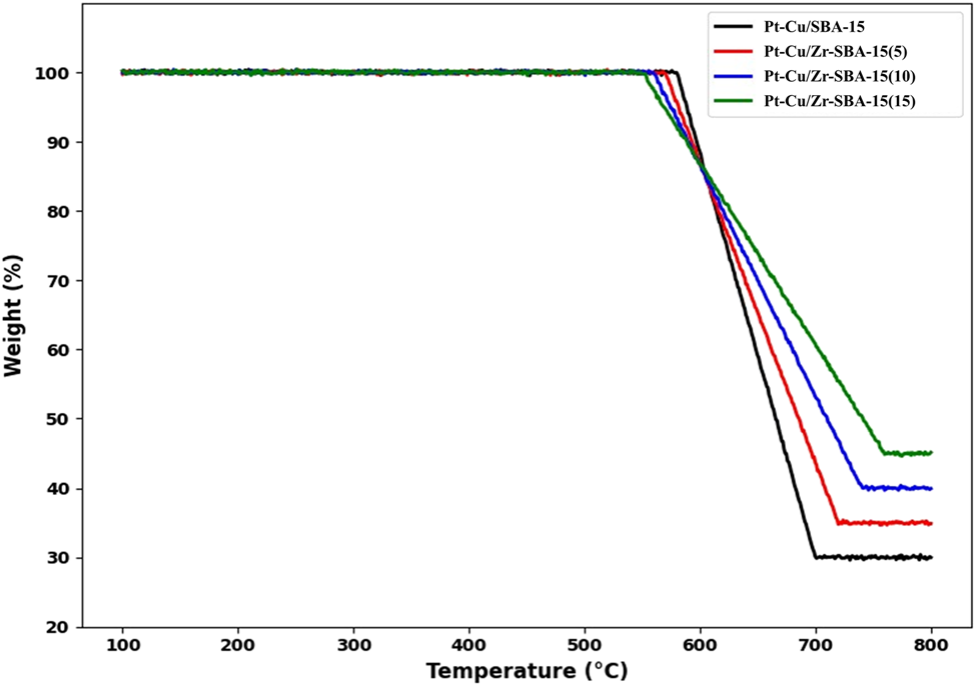
Thermogravimetric analysis (TGA) curves of (a) Pt-Cu/SBA-15, (b) Pt-Cu/Zr-SBA-15(5), (c) Pt-Cu/Zr-SBA-15(10), and (d) Pt-Cu/Zr-SBA-15(15).

Fig. 7 presents the thermogravimetric analysis (TGA) curves for Pt-Cu/SBA-15 and Pt-Cu/Zr-SBA-15 catalysts with varying Zr contents (5%, 10%, and 15%). The thermogravimetric profiles were carefully analyzed to evaluate the thermal stability of the catalysts and to estimate the extent of carbon deposition formed during the DRM reaction. The initial minor weight loss observed below approximately 200 °C can be attributed to the removal of physically adsorbed moisture and residual volatile species present on the catalyst surface. The gradual weight change at intermediate temperatures is mainly associated with the removal of surface hydroxyl groups and minor decomposition of residual organic fragments. The more pronounced weight loss occurring at higher temperatures is related to the oxidation of carbonaceous deposits formed during the reaction. The relatively smaller weight loss observed for the Zr-modified catalysts indicates a lower amount of carbon deposition compared with the unmodified Pt-Cu/SBA-15 catalyst. This behavior suggests that the incorporation of zirconium enhances catalyst stability and improves resistance to coke formation, likely due to stronger metal-support interactions and improved CO_2_ activation ability. All samples exhibit a relatively stable weight up to around 500 °C, exhibiting high thermal stability around this temperature. The substantial reduction in weight measured between 500 °C and 700 °C is primarily associated with thermal decomposition of residual organic species and structural hydroxyl groups, as well as possible dehydroxylation of the support framework. Among the samples, Pt-Cu/Zr-SBA-15(15%) shows the least weight loss, stabilizing around 45% at 800 °C, suggesting improved thermal stability with increasing Zr incorporation. In contrast, Pt-Cu/SBA-15 (without Zr) exhibits the highest weight loss, reaching approximately 30% residual weight, indicating lower thermal resistance. The intermediate samples, Pt-Cu/Zr-SBA-15(5%) and Pt-Cu/Zr-SBA-15(10%), show progressive improvement in thermal stability with increasing Zr content. The improved thermal stability of Pt-Cu/Zr-SBA-15 materials can be ascribed to the incorporation of zirconium, which enhances structural integrity and promotes stronger metal-support interactions. These results suggest that the addition of Zr into the SBA-15 framework contributes substantially to the thermal robustness of the catalyst. This enhanced thermal stability and reduced carbon deposition are consistent with the improved catalytic stability observed during the DRM reaction making it a promising candidate for high-temperature catalytic applications.

## 4. Catalytic behavior of bimetallic Pt-Cu supported on SBA-15 and Zr-modified SBA-15

**Fig. 8:**
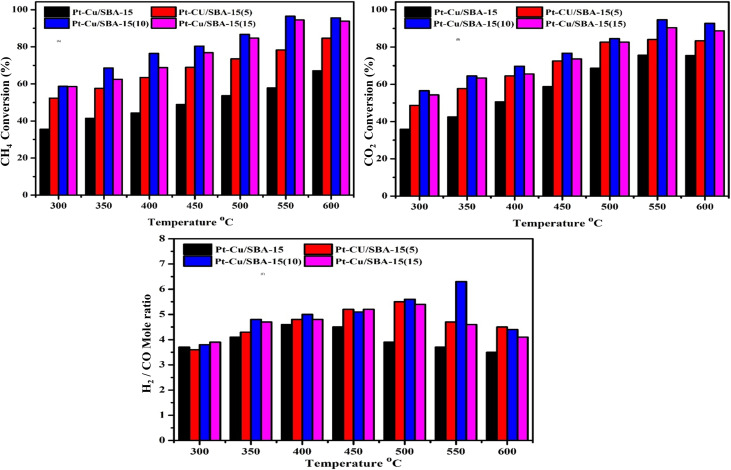
Effect of temperature on catalytic performance: (A) CH_4_ conversion, (B) CO_2_ conversion, and (C) H_2_/CO ratio over Pt-Cu/SBA-15 and Pt-Cu/Zr-SBA-15 catalysts. (Reactions were performed at atmospheric pressure with a CH_4_/CO_2_ ratio of 1 : 1, using N_2_ as a diluent, and a GHSV of 36 000 mL h^−1^ g cat^−1^).

The catalytic performance of Pt-Cu/SBA-15 and Pt-Cu/Zr-SBA-15(*x*) were evaluated for the DRM within a temperature range of 300–600 °C at atmospheric pressure. The performance trends are presented in Fig. 8(A–C). To better understand how efficiently these catalysts convert CH_4_ and CO_2_, and how this influences the H_2_/CO product ratio, additional tests were executed at a constant reaction temperature of 500 °C. This allowed for a clearer comparison between Pt-Cu/SBA-15 and its zirconium-modified counterpart under optimized reaction conditions.

### 4.1. Catalytic behavior of Pt-Cu/SBA-15 and Pt-Cu/Zr-SBA-15 at varying temperatures

The influence of temperature on catalytic properties of Pt–Cu-based systems—specifically Pt-Cu/SBA-15 and Zr-modified Pt-Cu/SBA-15 catalysts with varied zirconium loadings (5–15%) was examined under dry reforming of methane (DRM) conditions (CH_4_/CO_2_ = 1 : 1, GHSV = 36 000 mL h^−1^ g^−1^, atmospheric pressure, with N_2_ as the carrier gas). As shown in Fig. 8A, methane conversion and the H_2_/CO ratio exhibited a clear dependence on temperature, with overall performance improving steadily across the 300–600 °C range. At the lower end (300 °C), the unmodified Pt-Cu/SBA-15 catalyst gave the poorest activity, reaching only 35.5% CH_4_ conversion. In contrast, incorporating zirconium into the SBA-15 matrix considerably boosted activity. Notably, Pt-Cu/Zr-SBA-15(5) and Pt-Cu/Zr-SBA-15(10) delivered substantially higher conversions, ranging from 52.3% to 58.7% and 58.6%, respectively. As the temperature was elevated to 350 °C, the conversion rose across all catalysts, reaching 41.5% for Pt-Cu/SBA-15, 57.6–68.6% for Pt-Cu/Zr-SBA-15(5), and 62.5% for Pt-Cu/Zr-SBA-15(10). This trend continued at 400 °C and 450 °C, where the Pt-Cu/Zr-SBA-15(5) catalyst achieved conversions of 63.5% and 68.9%, while its replicate and the 10% Zr-loaded variant showed even higher conversions of 76.4–80.4% and 68.8–76.8%, respectively. At 500 °C, the performance of all catalysts further improved, with Pt-Cu/SBA-15 at 53.6%, Pt-Cu/Zr-SBA-15(5) in the range of 73.5–86.7%, and Pt-Cu/Zr-SBA-15(10) reaching 84.7%. Notably, the maximum catalytic activity was observed at 550 °C and 600 °C. At 550 °C, Pt-Cu/Zr-SBA-15(5) and Pt-Cu/Zr-SBA-15(10) catalysts exhibited remarkably high conversions of up to 96.5% and 94.5%, respectively, significantly outperforming the unmodified Pt-Cu/SBA-15 catalyst, which showed only 57.8% conversion. At 600 °C, although a slight decrease was observed for the Zr-containing samples (95.6% and 93.8%), these values remained substantially higher than the 67.1% achieved by the Pt-Cu/SBA-15 catalyst. Overall, the data indicate that embedding Zr into the SBA-15 framework substantially increases the catalytic activity of Pt–Cu catalysts in DRM, particularly at elevated temperatures. Among all, the Pt-Cu/Zr-SBA-15(5) catalyst demonstrated superior performance, achieving the highest CH_4_ conversion of 96.5% at 550 °C, likely due to optimal Zr loading that promotes strong metal-support interactions, improved dispersion of active sites, and enhanced thermal stability.^61^

Performance of Pt–Cu-based catalysts on SBA-15 and Zr-doped SBA-15 support was evaluated in the DRM under atmospheric pressure and a GHSV of 36 000 mL h^−1^ g_cat^−1^, with a CH_4_/CO_2_ ratio of 1 : 1 balanced with N_2_. The Fig. 8B shows CO_2_ conversion was measured across a temperature range from 300 to 600 °C, providing insights into the temperature dependence of catalytic activity and the influence of zirconium incorporation into the support matrix. The Pt-Cu/SBA-15 catalyst exhibited moderate catalytic activity, with CO_2_ conversion steadily increasing from 35.9% at 300 °C to a maximum of 75.6% at 550 °C, followed by a marginal decline to 75.5% at 600 °C. This trend indicates a positive temperature effect on activity, though the activity plateaued or slightly declined at the highest temperature, possibly due to sintering or diffusional limitations. In contrast, the Zr-incorporated catalysts, denoted as Pt-Cu/Zr-SBA-15(*x*) with varying zirconium contents (*x* = 5–15), showed significantly enhanced performance across all temperatures. Among the Zr-containing catalysts, Pt-Cu/Zr-SBA-15(5) demonstrated superior activity compared to the unmodified support, with CO_2_ conversion ranging from 48.7% at 300 °C to a peak of 84.0% at 550 °C. Catalyst with a higher Zr content, Pt-Cu/Zr-SBA-15(10), further improved activity, reaching the highest CO_2_ conversion of 94.6% at 550 °C. However, beyond this temperature, a slight decrease to 92.7% at 600 °C was observed, suggesting an optimal temperature window for catalytic performance. Overall, Zr-doping of the SBA-15 support considerably enhanced the catalytic efficiency of Pt–Cu catalysts in DRM, likely due to improved metal dispersion, stronger metal-support interactions, and increased surface acidity or basicity favorable for CO_2_ adsorption and activation. The performance hierarchy Pt-Cu/Zr-SBA-15(10) > Pt-Cu/Zr-SBA-15(15) > Pt-Cu/Zr-SBA-15(15) > Pt-Cu/SBA-15 confirms the beneficial role of Zr modification in tailoring support properties to maximize catalytic output, particularly at elevated temperatures.^62^

The performance of Pt–Cu-based catalysts on SBA-15 and Zr-modified SBA-15 supports was systematically evaluated in the DRM across a temperature range of 300 to 600 °C under atmospheric pressure, maintaining a CH_4_/CO_2_ molar ratio of 1 : 1 and a GHSV of 36 000 mL h^−1^ g_cat^−1^. The H_2_/CO product ratio was taken as a representative metric to assess the catalyst behavior considering both activity and selectivity are shown Fig. 8C. At 300 °C, the H_2_/CO ratio for all catalysts was below 4, indicating limited methane and carbon dioxide activation. Among them, Pt-Cu/Zr-SBA-15(15) showed the highest ratio (3.9), slightly outperforming the unmodified Pt-Cu/SBA-15 catalyst (3.7). As the operating temperature was elevated to 350 °C, a notable improvement in catalytic activity was observed, with Pt-Cu/Zr-SBA-15(10) exhibiting a significant rise to 4.8, suggesting that moderate Zr incorporation enhances reforming performance. Pt-Cu/Zr-SBA-15(5) and (15) also showed elevated ratios of 4.3 and 4.7, respectively, while Pt-Cu/SBA-15 trailed at 4.1. At 400 °C, the H_2_/CO ratios of the Zr-modified catalysts plateaued or marginally increased, reaching up to 5.0 for Pt-Cu/Zr-SBA-15(10), which retained the superior performance. In contrast, the Pt-Cu/SBA-15 catalyst maintained a slightly lower value of 4.6. Further increasing the temperature to 450 °C resulted in the highest observed H_2_/CO ratios for Pt-Cu/Zr-SBA-15(5) and (15) at 5.2, closely followed by (10) at 5.1, while Pt-Cu/SBA-15 remained unchanged or slightly decreased to 4.5, demonstrating the beneficial effect of Zr incorporation at this temperature range. Interestingly, at 500 °C, Pt-Cu/Zr-SBA-15(10) reached its maximum H_2_/CO ratio of 5.6, indicating optimal catalytic activity and possibly improved CH_4_ and CO_2_ conversion efficiency. Pt-Cu/Zr-SBA-15(5) and (15) followed closely with values of 5.5 and 5.4, respectively. However, Pt-Cu/SBA-15 exhibited a decline in performance with a ratio of 3.9, underscoring the superior thermal robustness of the Zr-promoted systems. At 550 °C, a divergence in catalyst behavior became apparent. Pt-Cu/Zr-SBA-15(10) continued to enhance its performance with a peak H_2_/CO ratio of 6.3 indicating strong resistance to sintering and possibly improved metal dispersion. In contrast, Pt-Cu/Zr-SBA-15(5) and (15) showed reduced ratios of 4.7 and 4.6, respectively, and Pt-Cu/SBA-15 continued its downward trend to 3.7. This suggests that excessive or insufficient Zr loading may compromise performance at high temperatures. At 600 °C, all catalysts experienced a decline in H_2_/CO ratio, potentially due to reverse water–gas shift reaction or catalyst deactivation. Pt-Cu/Zr-SBA-15(10) maintained a relatively higher ratio (4.4) compared to Pt-Cu/Zr-SBA-15(5) (4.5) and (15) (4.1), whereas Pt-Cu/SBA-15 declined further to 3.5. These results indicate that the embedding zirconium within the SBA-15 framework substantially enhances activity of catalyst and H_2_/CO selectivity, particularly at elevated temperatures, with an optimal Zr content identified around 10 wt%.

**Fig. 9:**
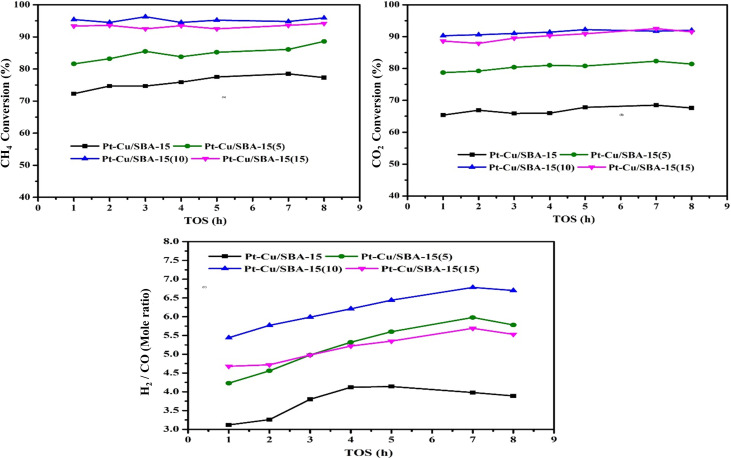
Time-on-stream performance at 550 °C: (A) CH_4_ conversion, (B) CO_2_ conversion, and (C) H_2_/CO ratio. (Conditions: CH_4_/CO_2_ = 1 : 1 with N_2_, GHSV = 36 000 mL h^−1^ g cat^−1^, atmospheric pressure).

The Pt–Cu catalysts supported on SBA-15 and Zr-incorporated SBA-15 were evaluated for catalytic performance with a range of Si-to-Zr molar ratios was evaluated during the DRM at 550 °C, under a GHSV of 36 000 mL h^−1^ g cat^−1^, using a CH_4_/CO_2_ ratio of 1 : 1 balanced with N_2_ at atmospheric pressure are shown in Fig. 9A. The time-on-stream performance, measured in terms of CH_4_ conversion, revealed distinct trends based on the support composition. The monometallic Pt-Cu/SBA-15 catalyst exhibited relatively lower and gradually increasing CH_4_ conversion values, ranging from 72.3% to a maximum of 78.5% over time, before slightly declining to the initial value. In contrast, the Pt-Cu/Zr-SBA-15 catalysts demonstrated significantly enhanced catalytic performance, with activity strongly influenced by the Zr content. Among the Zr-incorporated samples, Pt-Cu/Zr-SBA-15(10) showed the highest and most stable CH_4_ conversion throughout the duration of the test, maintaining values in the narrow range of 94.5–96.3%. This suggests an optimal Zr loading at the Si/Zr = 10 ratio that promotes superior activity, possibly as a result of enhanced dispersion of active sites and enhanced metal-support interaction. The Pt-Cu/Zr-SBA-15(15) catalyst also displayed high CH_4_ conversions, ranging between 92.5% and 94.2%, indicating good stability but a slight decline relative to the 10% Zr sample, which may result from excess Zr affecting the structural or electronic properties of the active sites. Interestingly, the Pt-Cu/Zr-SBA-15(5) catalyst, although superior to Pt-Cu/SBA-15, exhibited relatively lower CH_4_ conversions (81.6–88.6%) compared to its higher Zr-loaded counterparts, indicating that a lower Zr content is less effective in enhancing catalytic performance. These observations suggest that the embedding of Zr within the SBA-15 significantly improves the DRM activity of Pt–Cu catalysts, with an optimal effect observed at moderate Zr loading (Si/Zr = 10), balancing the structural benefits and active site accessibility.^63^

Performance of Pt–Cu catalysts anchored on SBA-15 and Zr-doped SBA-15 with varying Si/Zr ratios was evaluated for the DRM based on CO_2_ conversion data over time at 550 °C. The reaction conditions were maintained constant with a GHSV of 36 000 mL h^−1^ g_cat^−1^ and a 1 : 1 CH_4_/CO_2_ ratio in the presence of nitrogen under atmospheric pressure are shown in Fig. 9B. The baseline catalyst, Pt-Cu/SBA-15, exhibited relatively modest activity, with CO_2_ conversion values ranging narrowly between 65.4% and 68.5% over the 8-hour time-on-stream (TOS) evaluation. In contrast, the catalysts modified with zirconium showed significantly enhanced performance. Among the Zr-incorporated samples, Pt-Cu/Zr-SBA-15(10) demonstrated the highest and most stable CO_2_ conversion, ranging from 90.3% to 92.2%, indicating a superior catalytic efficiency and resilience during the prolonged reaction period. The Pt-Cu/Zr-SBA-15(15) variant also showed excellent activity, maintaining conversion levels between 87.9% and 92.5%, slightly fluctuating but remaining robust over time. Meanwhile, Pt-Cu/Zr-SBA-15(5) presented intermediate activity with conversion rates between 78.7% and 82.3%, outperforming the Zr-free counterpart but falling short of the higher-Zr-loaded samples. These results clearly demonstrate that the embedding of zirconium into the SBA-15 support substantially enhances the catalytic performance of Pt–Cu-based catalysts in DRM. Particularly, the optimized Si/Zr ratio in Pt-Cu/Zr-SBA-15(10) appears to offer the best balance between metal dispersion, support interaction, and catalytic stability, leading to high CO_2_ conversion efficiency.

Performance of the synthesized Pt–Cu catalysts loaded onto SBA-15 and Zr-SBA-15 with varying zirconium content was evaluated in terms of methane (CH_4_) conversion at 550 °C under a GHSV of 36 000 mL h^−1^ g cat^−1^ using a CH_4_/CO_2_ ratio of 1 : 1 with nitrogen at atmospheric pressure are shown in Fig. 9C. The Pt-Cu/SBA-15 catalyst exhibited relatively low CH_4_ conversion throughout the time-on-stream study, starting at 3.12% and gradually increasing to a peak of 4.14% before slightly declining. In contrast, the introduction of zirconium into the SBA-15 support significantly enhanced the performance of the catalyst. Specifically, the Pt-Cu/Zr-SBA-15(5) catalyst showed improved activity, reaching a maximum CH_4_ conversion of 5.98%. Further enhancement was observed with increased zirconium loading: Pt-Cu/Zr-SBA-15(10) exhibited the highest CH_4_ conversion, achieving a peak value of 6.78%, which remained relatively stable over the test duration. However, when the zirconium content was increased to the highest level in Pt-Cu/Zr-SBA-15(15), the conversion efficiency showed moderate performance compared to the 10% zirconium variant, peaking at 5.69%. These results highlight that incorporation of zirconium within SBA-15 supports improves CH_4_ conversion in DRM, with an optimal zirconium content around 10% delivering the best catalytic performance.

### 4.2. Comparative study

Evaluation of Pt-Cu/Zr-SBA-15 catalytic performance in DRM was evaluated and compared with various literature-reported catalysts are depicted in Table 4. The Pt-Cu/Zr-SBA-15 catalyst exhibited 82% CH_4_ conversion and 85% CO_2_ conversion with an H_2_/CO ratio of 0.98 at 550 °C over a 6-hour time-on-stream, demonstrating excellent stability and minimal coke formation. In contrast, conventional Ni/Al_2_O_3_ catalysts, despite delivering 65–70% CH_4_ conversion and 68–72% CO_2_ conversion at a higher temperature of 700 °C over 10 hours, suffered from high coking and rapid deactivation. Similarly, Ni–MgO offered moderate conversions (75% CH_4_, 76% CO_2_) with a comparable H_2_/CO ratio (0.9–1.0) but also displayed moderate coking at 700 °C for 12 hours. Rh/CeO_2_ performed better with 85–90% CH_4_ and 85–88% CO_2_ conversions at 650 °C for 30 hours, showing excellent stability and minimal carbon deposition. The Ni-Pt/KIT-5 catalyst achieved slightly higher conversions (88% CH_4_, 89% CO_2_) with an H_2_/CO ratio of 1.05 at 600 °C over 25 hours, benefiting from the NiPtO_2_ spinel phase that hindered sintering. Co–Ni/Al_2_O_3_ and Pt/Ce–ZrO_2_ systems presented moderate to high performance, with the latter offering high thermal stability. Ni–Cu/La_2_O_3_ demonstrated improved coke resistance due to the presence of Cu, delivering 78% CH_4_ and 80% CO_2_ conversions at 650 °C for 15 hours. Overall, the Pt-Cu/Zr-SBA-15 catalyst proved to be highly competitive, offering a good balance of conversion efficiency, syngas quality, and long-term stability at relatively lower operating temperatures.

**Table 4:** Comparative performance of Pt-Cu/Zr-SBA-15 with reported DRM catalysts

S. no	Catalyst system	CH_4_ conversion (%)	CO_2_ conversion (%)	H_2_/CO ratio	Temp (°C)	Time-on-stream (h)	Coke resistance/stability	Ref.
1	Pt-Cu/Zr-SBA-15	94.3	91.4	1 : 1	550	6	Excellent stability, low coke	Present work
2	Ni/Al_2_O_3_	65–70	68–72	∼1.0	700	10	High coking, fast deactivation	64
3	Ni–MgO	75	76	0.9–1.0	700	12	Moderate coking	65
4	Rh/CeO_2_	85–90	85–88	∼1.0	650	30	Very stable, low carbon formation	66
5	Ni–Pt/KIT-5	88	89	1.05	600	25	Excellent, NiPtO_2_ prevents sintering	67
6	Co–Ni/Al_2_O_3_	70	68	0.95	700	8	Moderate coking	68
7	Pt/Ce–ZrO_2_	80	81	1.0	600	24	High thermal stability	69
8	Ni–Cu/La_2_O_3_	78	80	0.92	650	15	Improved coke resistance *via* Cu	70

### 4.3. Mechanistic considerations

**Fig. 10:**
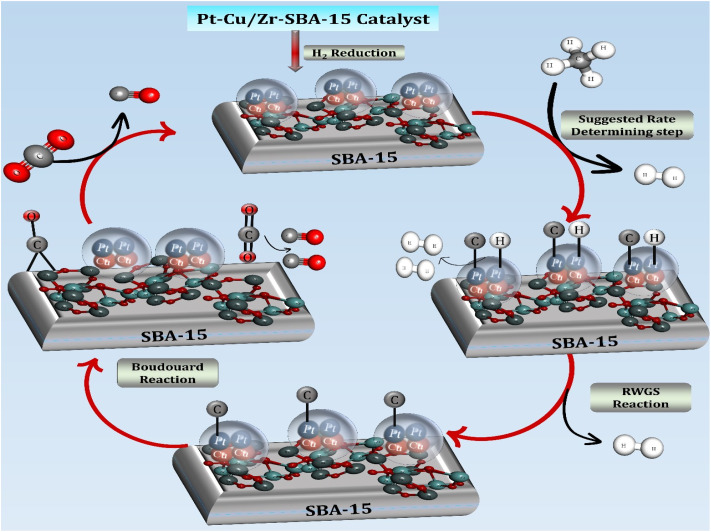
Proposed reaction mechanism for DRM over Pt-Cu/Zr-SBA-15 catalysts.

The mechanistic interpretation presented in this work is primarily based on the experimental characterization results together with previously reported mechanistic studies on DRM catalysts are shown in Fig. 10. Recent investigations have shown that the DRM reaction proceeds through a sequence of elementary surface reactions involving the activation of methane and carbon dioxide on catalytic active sites. Methane activation typically occurs *via* stepwise C–H bond cleavage on metallic sites, producing surface intermediates such as 
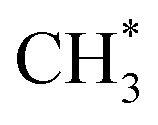
, 
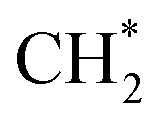
, CH*, and C*, along with adsorbed hydrogen species. In parallel, CO_2_ molecules adsorb on metal or metal-support interfacial sites and undergo dissociation to form CO* and reactive oxygen species (O* or OH*). These oxygen-containing species play a critical role in removing surface carbon species through oxidation reactions, thereby regenerating active catalytic sites and suppressing coke accumulation. Such a bifunctional reaction pathway highlights the importance of cooperative interactions between metal particles and the catalyst support in promoting both CH_4_ activation and CO_2_ dissociation.^71^ In the present Pt-Cu/Zr-SBA-15 catalytic system, methane activation is considered to occur predominantly on metallic Cu sites where the C–H bond cleavage generates CH_*x*_ intermediates and surface hydrogen species. Meanwhile, platinum sites facilitate the adsorption and dissociation of CO_2_, generating reactive oxygen species that participate in the oxidation of carbonaceous intermediates formed during methane decomposition. The incorporation of zirconium into the SBA-15 framework introduces Lewis acidic Zr^4+^ sites and strengthens metal-support interactions, which enhance CO_2_ adsorption and activation at the metal-support interface. The reactive oxygen species formed during CO_2_ activation can subsequently react with carbon intermediates to produce CO, thereby preventing carbon accumulation on the catalyst surface. Simultaneously, the hydrogen atoms generated during methane decomposition recombine to form molecular hydrogen (H_2_), contributing to the overall syngas production. Consequently, the synergistic interaction between Pt, Cu, and the Zr-modified SBA-15 support promotes efficient activation of both reactants, enhances resistance to coke formation, and improves the overall catalytic performance during the DRM reaction.

### 4.4. Molecular docking analyses

Fig. S2–S6 provide detailed molecular docking analyses of the Zr-SBA-15-Cu–Pt catalyst with the reactants and products of the dry reforming reaction. These include interactions with CH_4_ (S1), CO_2_ (S2), CO (S3), and H_2_ (S4), as well as the coordination between the catalyst support and active metals (S5). The figures illustrate hydrogen bonding, metal-acceptor interactions, and unfavorable contacts, supporting the mechanistic insights discussed in the main text. All docking results were generated using Hex software and visualized in BIOVIA Discovery Studio.

## 5. Conclusion

We developed a series of bimetallic Pt–Cu catalysts anchored on Zr-modified SBA-15 with varying Si/Zr ratios and evaluated their performance in the DRM. Structural analyses provided clear insights into how Zr incorporation influenced the catalyst framework. XRD and TEM confirmed that the SBA-15 retained its highly ordered mesoporous structure, while at higher Zr contents, anatase ZrO_2_ domains began to emerge. The copper component was mainly detected as crystalline CuO, consistent with its characteristic diffraction peaks. FT-IR spectra further verified the successful substitution of Zr^4+^ into the silica matrix, while NH_3_-TPD results revealed that Zr addition substantially increased surface acidity. This enhanced acidity was pivotal in facilitating better dispersion of Pt and Cu nanoparticles across the support. Among the synthesized catalysts, the Pt-Cu/Zr-SBA-15 possessing a Si to Zr ratio of 10 displayed the richest distribution of both weak and strong acid sites. Under optimized operating parameters, all catalysts including the unmodified SBA-15-supported version achieved high CH_4_ and CO_2_ conversions. However, the Si/Zr = 10 catalyst consistently outperformed the others, delivering superior activity and long-term stability. Remarkably, it maintained its performance even at 600 °C, demonstrating strong resistance to carbon deposition. The improved performance observed for the Si/Zr = 10 catalyst could be attributed to more intimate metal-support interactions, stronger acidity, and finer dispersion of metal particles due to Zr^4+^ incorporation. Overall, these findings clearly show that introducing Zr into the SBA-15 framework not only tailors the textural and acidic properties of the support but also enhances the synergistic interplay between Pt and Cu. As a result, the Pt-Cu/Zr-SBA-15 series emerges as a highly promising platform for DRM, combining high catalytic activity with durability and excellent resistance to coking.

## 6. Future studies

Perform DFT and kinetic studies to elucidate the detailed reaction mechanism and energy barriers for CH_4_ and CO_2_ activation. Optimize Si/Zr ratios further to balance surface acidity, metal dispersion, and structural stability for enhanced performance. Conduct long-term DRM tests under harsher conditions to evaluate carbon deposition and catalyst durability. Investigate different Pt/Cu ratios and alternative promoters to improve synergistic effects and CO_2_ activation. Apply *in situ* characterization techniques to monitor surface species and metal states during the reaction.

## Conflicts of interest

The authors declare no conflict of interest.

## Supplementary Material

RA-016-D6RA02279K-s001

## Data Availability

The data that support the findings of this study are available from the corresponding author upon reasonable request. Supplementary information (SI) is available. See DOI: https://doi.org/10.1039/d6ra02279k.
