# Supplementary material for: Effect of Si/Zr ratio on the catalytic behavior of Pt-Cu/Zr-SBA-15 in continuous methane dry reforming
Source: RSC Adv. 2026 Apr 20;16(22):20347–63. doi: 10.1039/d6ra02279k (PMC13093882; doi:10.1039/d6ra02279k)
Supplement: RA-016-D6RA02279K-s001 [file RA-016-D6RA02279K-s001.pdf]

Supplementary Information (SI) for RSC Advances.
This journal is © The Royal Society of Chemistry 2026

High-Resolution Scanning Electron Microscopy.

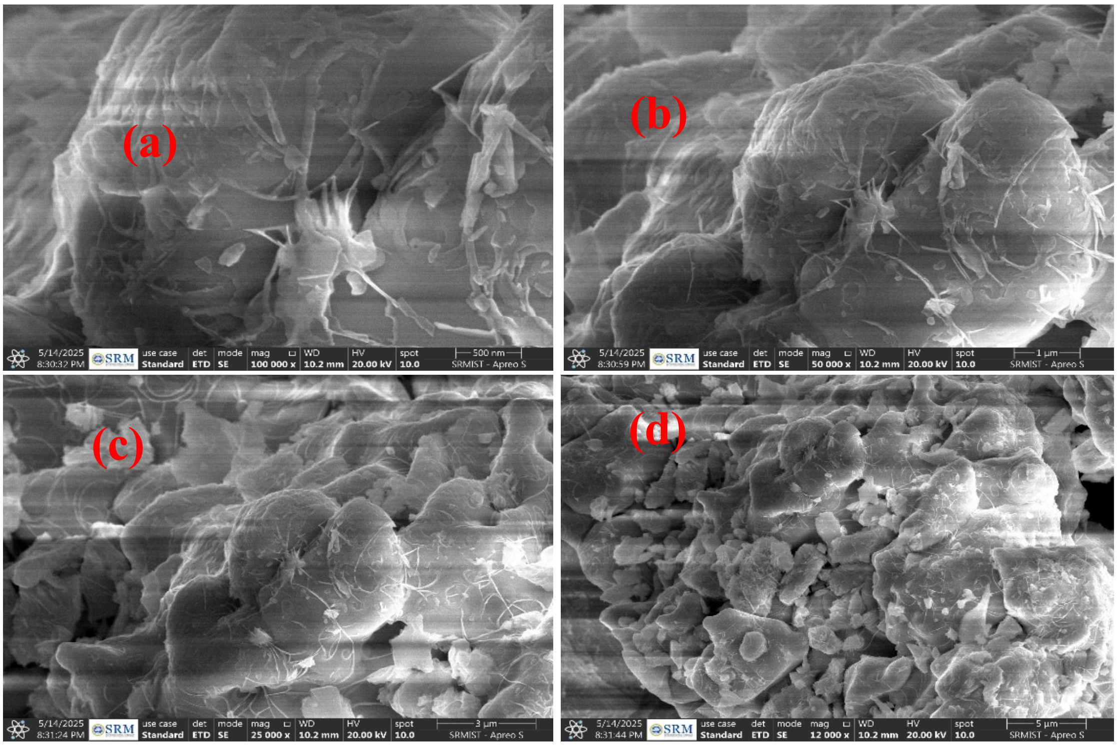

Figure.S1. HR-SEM Images of a) Pt-Cu/SBA-15**, b) Pt-Cu/Zr-SBA-15(5), c) Pt-Cu/Zr-SBA-15(10), and d) Pt-Cu/Zr-SBA-15(15).**

Molecular Docking Analyses

Molecular docking studies for the complexes were carried out using Hex docking software to evaluate the interaction energies and geometries between the Zr–SBA-15–Cu–Pt catalyst and reactant/product molecules (CH_4, CO_2, CO, and H_2). The resulting docking poses and non-covalent interactions, including hydrogen bonds and metal–acceptor contacts, were further visualized and analyzed using BIOVIA Discovery Studio, allowing detailed inspection of the adsorption sites and interaction networks.

Zr–SBA-15–Cu–Pt catalyst Molecular Docking Studies

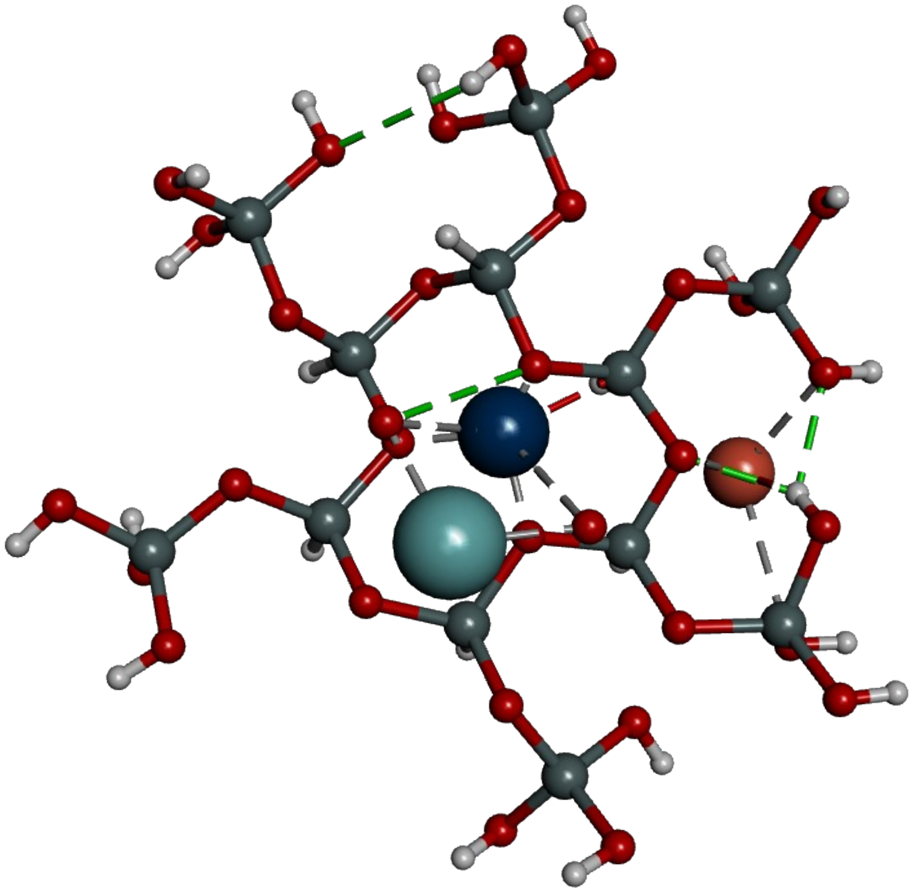

Figure S2. Interaction profile of the Zr–SBA-16–Cu–Pt catalyst illustrating the coordination between Zr-modified SBA-16 support and Cu/Pt active metal sites

The interaction profile of the Zr–SBA-15–Cu–Pt catalyst (E-total = −30.97 kcal/mol) indicates a moderately strong and well-balanced adsorption of the ligand on the catalyst surface, governed by a combination of hydrogen bonding and metal–acceptor interactions are shown in **Figure S2.** The presence of multiple conventional hydrogen bonds, particularly H···O interactions in the range of 2.04–2.14 Å, reflects strong stabilization of the adsorbed species, while longer contacts (~2.93–3.29 Å) contribute to weaker secondary stabilization and proper orientation of the molecule on the surface. The Zr–O interactions (~2.22 Å) confirm the role

of zirconium as a Lewis acidic center, enhancing the polarization of oxygen-containing groups and facilitating adsorption. In comparison, Cu–O interactions (2.60–3.18 Å) are relatively weaker, suggesting a moderate coordination environment that can support redox activity without excessively stabilizing intermediates. Notably, the Pt–O interactions exhibit the shortest distances (1.22–1.86 Å), indicating very strong chemisorption and identifying Pt as the primary active site responsible for strong binding and activation of reactant molecules. The coexistence of strong Pt–O bonding, moderate Cu–O coordination, and Zr-induced acidity demonstrates a synergistic effect within the catalyst, enabling efficient adsorption, activation, and stabilization of intermediates. Overall, the interaction energy and bonding characteristics suggest that the catalyst provides an optimal balance between stability and reactivity, which is essential for effective catalytic performance (**Figure S2.**).

Zr–SBA-15–Cu–Pt catalyst - Methane

The interaction analysis of methane on the Zr–SBA-15–Cu–Pt catalyst, despite exhibiting an overall interaction energy of E-total = −66.72 kcal/mol, indicates that there is no direct or specific interaction between CH_4 and the active sites of the catalyst **Figure S3.**. The significantly negative energy value suggests a globally stabilized system; however, the absence of identifiable C–H···surface or metal–C interactions confirms that methane itself does not actively participate in strong binding with the catalyst. The observed hydrogen bonding interactions (H···O and O···O) are not associated with methane but rather arise from surface hydroxyl groups or oxygen-containing species within the catalyst framework. Similarly, the Zr–O (~2.22 Å), Cu–O (2.60–3.18 Å), and Pt–O (1.22–1.86 Å) metal–acceptor interactions indicate strong coordination between the metal centers and oxygen atoms, further confirming that the interaction network is dominated by catalyst–oxygen species rather than methane adsorption. Therefore, although the total interaction energy is relatively high, it reflects overall system stabilization rather than direct methane activation. This suggests that CH_4 remains weakly adsorbed or essentially non-interacting, and its activation in the dry reforming process likely requires higher thermal energy or proceeds via indirect pathways involving reactive oxygen species rather than direct metal–methane bonding.

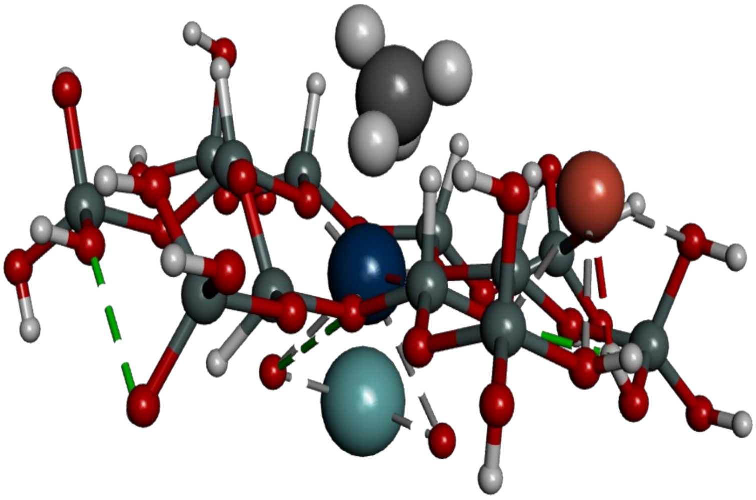

Figure S3. Interaction analysis of methane (CH_4) with the Zr–SBA-16–Cu–Pt catalyst showing the absence of direct CH_4–metal interactions

Zr–SBA-15–Cu–Pt catalyst – Carbon-di-oxide

The interaction analysis of carbon dioxide on the Zr–SBA-15–Cu–Pt catalyst (E-total = −66.72 kcal/mol) indicates strong overall stabilization of the adsorbate–catalyst system, primarily governed by metal–oxygen coordination and secondary hydrogen bonding interactions **Figure S4.** The presence of a single prominent H···O hydrogen bond (~2.04 Å), along with additional weaker H···O contacts (2.13–2.93 Å) and an O···O interaction (~3.29 Å), suggests limited but supportive hydrogen bonding that aids in orienting and stabilizing the CO_2 molecule on the catalyst surface. The Zr–O interactions (~2.22 Å) confirm the involvement of zirconium as a Lewis acidic site, which facilitates polarization of the CO_2 molecule and enhances its susceptibility to activation. Cu–O interactions in the range of 2.60–3.18 Å indicate moderate coordination, contributing to electronic interactions without excessively stabilizing the adsorbate. Notably, the Pt–O interactions exhibit very short distances (1.22–1.86 Å), reflecting strong chemisorption and indicating that Pt serves as the primary active site for CO_2 adsorption and activation. The longer Pt–O contacts (~2.96–3.29 Å) represent weaker secondary interactions that further stabilize the adsorbed configuration. Overall, despite the limited hydrogen bonding contribution, the highly negative interaction energy is dominated by strong metal–oxygen interactions, particularly from Pt and Zr, which promote effective CO_2 activation. This suggests that the catalyst provides a favorable surface for CO_2 adsorption and polarization, an essential step in the dry reforming reaction pathway.

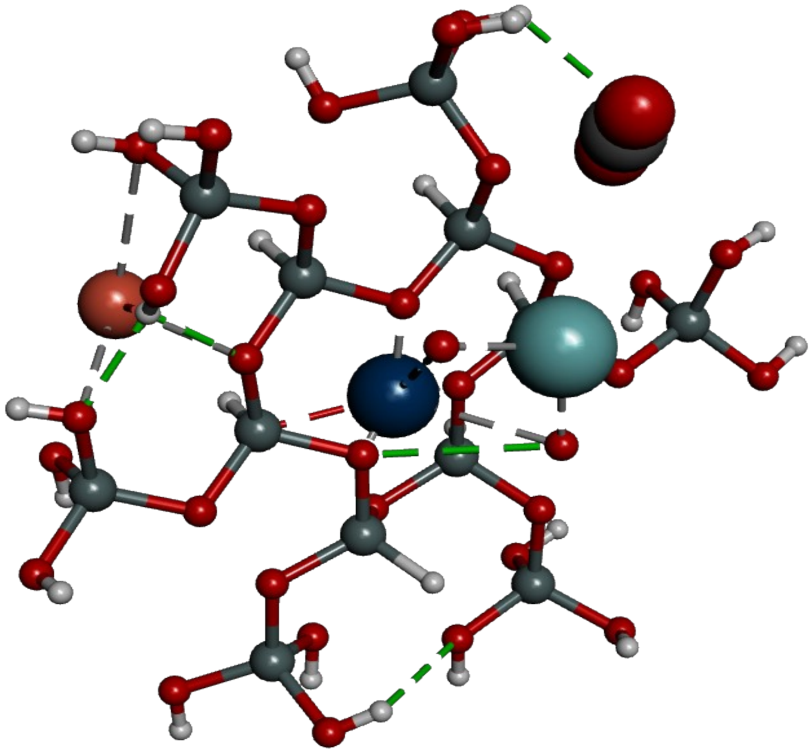

Figure S4. Interaction analysis of carbon dioxide (CO_2) with the Zr–SBA-16–Cu–Pt catalyst showing dominant metal–oxygen coordination (Pt–O, Zr–O, and Cu–O interactions)

Zr–SBA-15–Cu–Pt catalyst – Carbon-monoxide

The interaction analysis of carbon monoxide adsorption on the Zr–SBA-15–Cu–Pt catalyst shows a strongly stabilized system with an overall interaction energy of **E-total = −65.41 kcal/mol**, indicating favorable adsorption on the catalyst surface are shown in **Figure S5.**.. The interaction network is dominated by multiple conventional hydrogen bonds, including strong H···O contacts (2.04–2.14 Å) and weaker interactions (~2.93 Å), along with an O···O interaction (~3.29 Å), which together contribute to secondary stabilization and proper alignment of the adsorbed species. Significant metal–oxygen coordination is observed, with Zr–O interactions (~2.22 Å) confirming the presence of Lewis acidic sites, and Cu–O interactions (2.60–3.18 Å) indicating moderate binding that supports electronic interactions. The Pt–O interactions exhibit very short distances (1.22–1.86 Å), reflecting strong chemisorption and identifying Pt as the primary active site responsible for CO adsorption. In addition to these stabilizing interactions, a small number of unfavorable contacts, such as Si–Pt (2.36 Å), H–C (1.98 Å), Pt–C (2.23 Å), and H–Cu (2.37 Å), are observed, likely arising from steric constraints or non-ideal geometry of adsorption. However, these unfavorable interactions are minimal compared to the dominant stabilizing forces and therefore do not

significantly affect the overall adsorption stability. The highly negative interaction energy, combined with strong Pt-centered coordination and supportive Zr and Cu interactions, suggests that CO is effectively adsorbed and stabilized on the catalyst surface, facilitating its role as an intermediate in the dry reforming reaction.

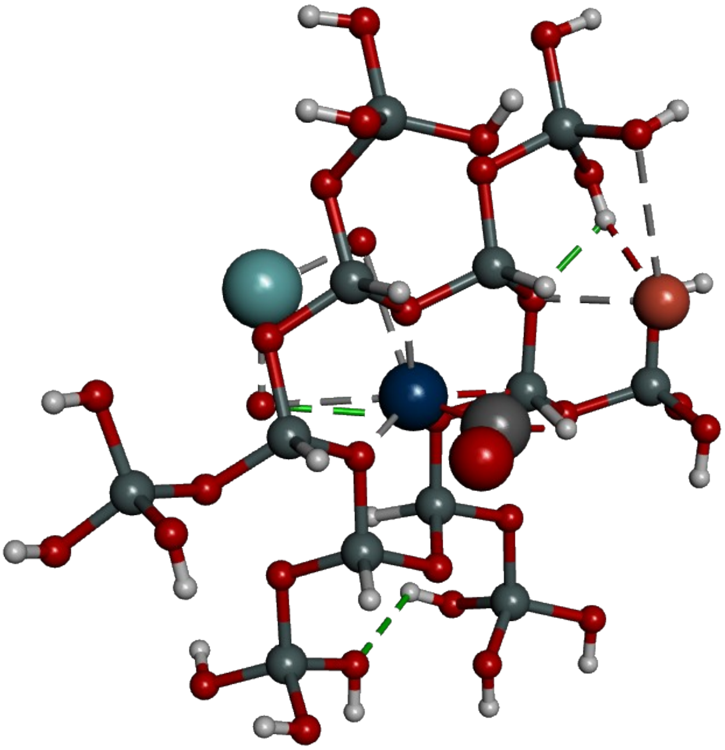

Figure S5. Interaction analysis of carbon monoxide (CO) with the Zr–SBA-15–Cu–Pt catalyst showing strong Pt-centered metal–oxygen coordination, supported by Zr and Cu interactions

Zr–SBA-15–Cu–Pt catalyst – Hydrogen

The interaction analysis of hydrogen (H_2) on the Zr–SBA-15–Cu–Pt catalyst clearly indicates an absence of favorable adsorption, as reflected by the overall interaction energy of **E-total =** 0 kcal/mol are shown **Figure S6.**. The system is characterized exclusively by unfavourable interactions, including multiple short O···H contacts (1.29–1.57 Å), Si–H (~2.25 Å), extremely short Pt–H distances (~0.45–0.50 Å), H–H (~0.71 Å), and H–Cu (~2.37 Å), all of which correspond to steric clashes or "unfavorable bump" interactions. These non-physical proximities suggest strong repulsion between hydrogen and the catalyst surface, preventing the formation of any stable adsorption geometry. Although hydrogen-bond-like H···O interactions appear in the dataset, they are not associated with molecular H_2 but rather originate from surface hydroxyl or framework species. Therefore, the interaction profile confirms that hydrogen does not participate in meaningful binding with the active sites (Zr, Cu, or Pt) and

remains non-interacting. This lack of adsorption is consistent with the role of H_2 as a final product in the dry reforming reaction, facilitating its easy desorption from the catalyst surface and preventing site blockage, thereby supporting continuous catalytic turnover.

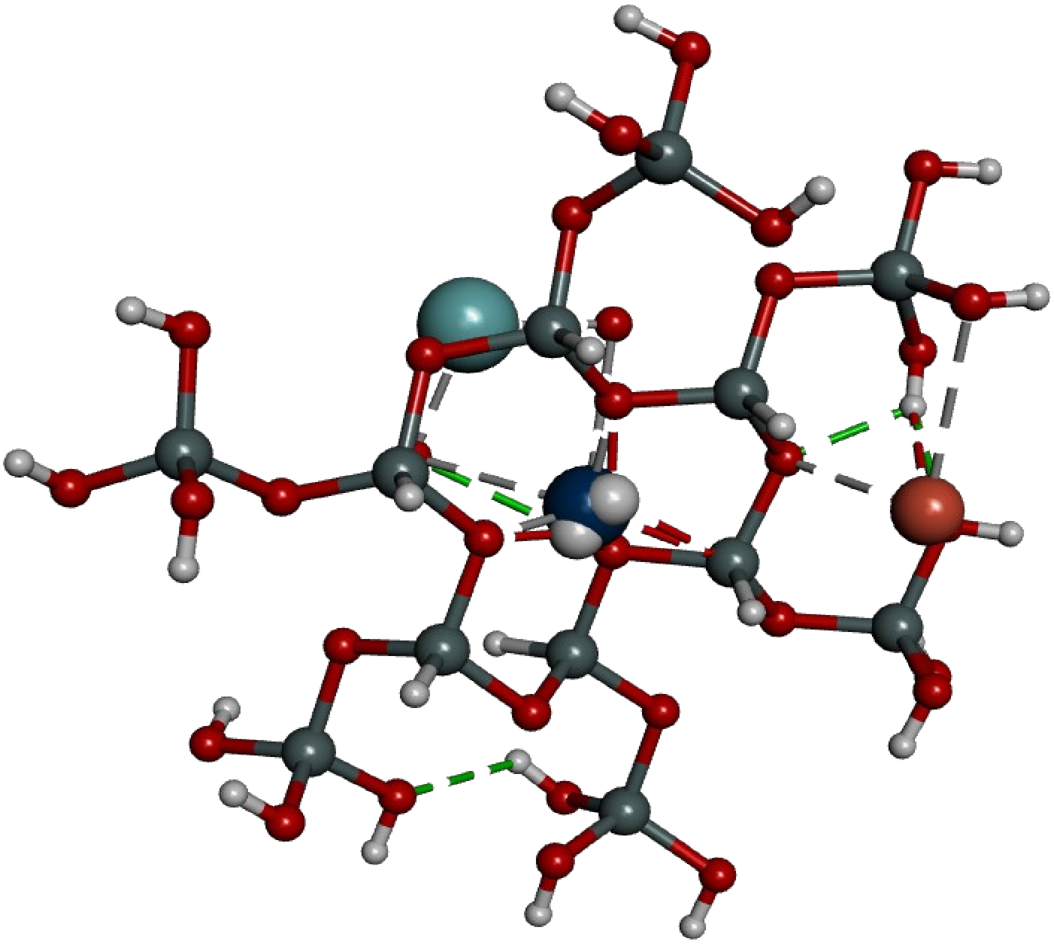

Figure S6. Interaction analysis of hydrogen (H_2) with the Zr–SBA-15–Cu–Pt catalyst showing exclusively unfavorable steric interactions and no significant adsorption

Overall Docking Interpretation of Zr–SBA-15–Cu–Pt Catalyst for Dry Reforming of Methane

The overall docking interpretation for the dry reforming of methane (DRM) over the Zr–SBA-15–Cu–Pt catalyst demonstrates a well-balanced catalytic system in which each reactant and product exhibits distinct interaction behavior, supporting an efficient reaction pathway. Carbon dioxide shows strong adsorption with a highly negative interaction energy (E-total ≈ −66.72 kcal/mol), dominated by very strong Pt–O interactions (1.22–1.86 Å) and supported by Zr–O coordination (~2.22 Å) and moderate Cu–O interactions, indicating effective CO_2 activation through polarization and chemisorption at Pt sites. In contrast, methane does not exhibit direct or significant interaction with the catalyst surface despite a high overall stabilization energy,

confirming that CH_4 remains largely non-interacting and requires higher thermal energy for C–H bond activation, rather than strong adsorption-driven activation.

The intermediate carbon monoxide displays strong adsorption (E-total ≈ −65.41 kcal/mol), primarily governed by Pt-centered coordination and supported by Zr and Cu interactions, with only minimal unfavourable contacts, suggesting stable intermediate formation without excessive surface poisoning. On the other hand, hydrogen (H_2) shows no favorable interaction (E-total = 0 kcal/mol) and is characterized only by unfavourable steric interactions, indicating that it does not bind to the catalyst surface and is readily desorbed after formation. Collectively, these results reveal a synergistic catalytic mechanism in which Pt acts as the primary active site for CO_2 activation and intermediate stabilization, Zr enhances surface acidity and promotes oxygen activation, and Cu contributes to moderate electronic interactions and redox behavior. The catalyst exhibits strong adsorption for CO_2 and CO, negligible interaction with CH_4, and complete non-adsorption of H_2, thereby ensuring efficient reactant activation, controlled intermediate stability, and easy product desorption. This balance between adsorption and desorption is critical for sustained catalytic performance and confirms the suitability of the Zr–SBA-15–Cu–Pt system for dry reforming reactions.
